# Evaluation of Linear Growth at Higher Altitudes

**DOI:** 10.1001/jamapediatrics.2020.2386

**Published:** 2020-08-24

**Authors:** Kaleab Baye, Kalle Hirvonen

**Affiliations:** 1 Addis Ababa University, College of Natural and Computational Sciences, Center for Food Science and Nutrition, Addis Ababa, Ethiopia; 2 International Food Policy Research Institute, Development Strategy and Governance Division, Addis Ababa, Ethiopia

## Abstract

**Importance:**

Irrespective of their genetic makeup, children living in an ideal home environment that supports healthy growth have similar growth potential. However, whether this potential is true for children residing at higher altitudes remains unknown.

**Objective:**

To investigate whether altitude is associated with increased risk of linear growth faltering and evaluate the implications associated with the use of the 2006 World Health Organization growth standards, which have not been validated for populations residing 1500 m above sea level.

**Design, Settings, and Participants:**

Analysis of 133 nationally representative demographic and health cross-sectional surveys administered in 59 low- and middle-income countries using local polynomial and multivariate regression was conducted. A total of 964 299 height records from 96 552 clusters at altitudes ranging from −372 to 5951 m above sea level were included. Demographic and Health Surveys were conducted between 1992 and 2018.

**Exposures:**

Residence at higher altitudes, above and below 1500 m above sea level, and in ideal home environments (eg, access to safe water, sanitation, and health care).

**Main Outcomes and Measures:**

The primary outcome was child linear growth deficits expressed in length-for-age/height-for-age *z *scores (HAZ). Associations between altitude and height among all children and those residing in ideal home environments were assessed. Child growth trajectories above and below 1500 m above sea level were compared and the altitude-mediated height deficits were estimated using multivariable linear regression.

**Results:**

In 2010, a total of 842 million people in the global population (approximately 12%) lived 1500 m above sea level or higher, with 67% in Asia and Africa. Eleven percent of the sample was children who resided 1500 m above sea level or higher. These children were born at shorter length and remained on a lower growth trajectory than children residing in areas less than 1500 m above sea level. The negative association between altitude and HAZ was approximately linear through most part of the altitude distribution, indicating no clear threshold for an abrupt decrease in HAZ. A 1000-m above sea level increase in altitude was associated with a 0.163-unit (95% CI, −0.205 to −0.120 units) decrease in HAZ after adjusting for common risk factors using multivariable linear regressions. The HAZ distribution of children residing in ideal home environments was similar to the 2006 World Health Organization HAZ distribution, but only up to 500 m above sea level.

**Conclusions and Relevance:**

The findings of this study suggest that residing at a higher altitude may be associated with child growth slowing even for children living in ideal home environments. Interventions addressing altitude-mediated growth restrictions during pregnancy and early childhood should be identified and implemented.

## Key Points

**Question:**

Do children residing at higher altitudes have systematically different linear growth trajectories?

**Findings:**

In this cross-sectional study including 964 299 children aged 0 to 59 months, those residing at higher altitudes were, on average, born at a shorter length and remained on a lower growth trajectory than children residing at lower altitudes. This altitude-mediated growth difference remained statistically and biologically significant after controlling for potential confounding factors or when the sample was restricted to children living in ideal home environments.

**Meaning:**

The findings of this study suggest that specific attention and health care guidance are needed for the management of pregnancies and early child development in high-altitude settings; altitude-specific adjustments to 2006 World Health Organization growth standards are not recommended.

## Teaser

This cross-sectional study examines the growth trajectories of children born at higher altitudes.

## Introduction

Reducing child undernutrition has become one of the most important global objectives.^1,2^ Progress is routinely measured using anthropometric indexes, such as height-for-age *z* scores (HAZ) that compare child height with children whose physiologic needs are met and are living in environments that support healthy growth. Height-for-age *z* score measures these differences in terms of SDs, with negative values indicating that a child's height is below the height of the median child in a healthy, well-nourished reference population of the same age and sex. Children are considered to be chronically undernourished (stunted) if their HAZ is below −2.

Stunting affects approximately 155 million children (23%) younger than 5 years globally^3^ and is associated with an increased risk of mortality, cognitive deficits, and metabolic developmental impairments that predispose them to chronic diseases in later life.^4,5^ While the causes of stunting are multiple and complex, standard of living and access to basic needs, such as adequate nutrition and health care, are underlying and immediate causes.^6^ Child growth is regularly monitored through national surveys, such as the Demographic and Health Surveys (DHS) to measure progress toward the Sustainable Development Goals (SDG 2.2) to end all forms of malnutrition by 2030 and the World Health Organization (WHO) Global Nutrition Target that calls for 40% reduction in stunting by 2025.^1,2^

Global comparisons and child growth tracking rely on the assumption that children living in an ideal home environment that promotes adequate growth have the same growth potential, irrespective of their genetic makeup. This finding of similar child growth potential, regardless of genetic makeup, was first supported by the WHO Multicentre Growth Reference Study that reported how children (n = 8440) from affluent families but widely differing ethnic backgrounds and cultural settings had a similar growth pattern during the first 5 years of life.^7^ The understanding that all children have the same growth potential when their physiologic needs are met and the environments support healthy growth led to the development of a prescriptive growth standard that has now been universally adopted. With the DHS collecting data on child growth from multiple countries, it has become possible to replicate the findings of the Multicentre Growth Reference Study using data from 169 DHS surveys from 63 countries.^8^

However, among the exclusion criteria used in selecting sites for the Multicentre Growth Reference Study was residence at a high altitude: 1500 m or more above sea level.^9,10^ This decision was informed by the study by Yip et al^11^ that showed impaired growth among children living at high-altitude sites in the US.^10^ However, this study did not account for potential confounding factors. Moreover, the International Fetal and Newborn Growth Consortium for the 21st Century (also known as Intergrowth-21st) similarly excluded sites 1500 m or more above sea level when developing standards for fetal and neonatal growth.^12^ It remains unclear whether these widely used growth standards are applicable to populations living above the 1500 m above sea level threshold. The relevance and applicability of the growth standards at higher altitude depends on whether high-altitude residence per se is a risk factor for growth faltering or whether these growth deficits are manifestations of poorer living conditions that may be more pronounced at high altitudes.^13,14,15^ To our knowledge, there is no information on whether children in ideal home environments in low- and middle-income countries who reside at high altitudes (eg, ≥1500 m above sea level) have the same growth potential as those living at lower altitudes. This gap in knowledge remains despite a nonnegligible proportion of the global population (approximately 12%) residing at altitudes 1500 m or more above sea level and the WHO growth standards are being applied in these areas without discretion. Given that stunting is now used as an indicator to track progress on global nutrition targets and the sustainable development goals, it is necessary to revisit the association between altitude and child growth. Therefore, in this study we investigated whether altitude is associated with increased risk of linear growth faltering and evaluated whether the prescriptive WHO growth standards can apply to children residing at higher altitudes.

## Methods

While researchers have used different definitions for high altitude,^16^ we used the 1500 m above sea level threshold because sites located above this altitude were not considered eligible in the Multicentre Growth Reference Study. The analysis took place in stages. Surveys were conducted between 1992 and 2018. We first estimated the number of people residing 1500 m above sea level in each country. We then compared child growth trajectories at lower than 1500 m above sea level and 1500 m or more above sea level altitudes using local polynomial regression methods. Previous literature has attributed high-altitude growth deficits to poorer nutrition, health, and socioeconomic conditions at high-altitude localities.^13,17^ Considering these issues, we accounted for the role of confounding factors in 3 ways. First, we used linear regression methods to quantify the child height deficit associated with altitude after adjusting for confounding factors. Second, we used the same multivariable regression models to assess how altitude is associated with immediate causes of malnutrition: diets and disease.^4^ Third, we restricted the sample to children who resided in ideal home environments^8^ and used regression methods to assess the association between altitude and child height within this sample. The protocols and questionnaires of DHS surveys have been reviewed and approved by ICF institutional review board and the institutional review boards of the host countries. This study followed the Strengthening the Reporting of Observational Studies in Epidemiology (STROBE) reporting guideline for cross-sectional studies.

The country-level population data disaggregated by altitude were based on data from the Center for International Earth Science Information Network (CIESIN), Columbia University.^18^ We used nationally representative and publicly available cross-sectional DHS survey data from 59 low- and middle-income countries. We used all available DHS surveys that collected anthropometric measures for children younger than 5 years and restricted the analysis to surveys that recorded the altitude of the survey cluster or the global positioning system coordinates of the cluster that permitted us to obtain the altitude from external sources. A cluster was equivalent to a village in rural areas or a neighborhood in urban areas. Linear growth (faltering) was expressed in HAZ that measures the distance in height to the median child in the 2006 WHO growth standard.^7^ For convenience, we used the term *HAZ* to refer to both length for age (<24 months) and height for age (≥24 months). Children with biologically implausible height measures (HAZ <−6 or HAZ >6) were excluded from the analysis. A total of 964 299 height records (51% boys, 49% girls) from 96 552 clusters from 133 surveys administered in 59 countries were used (eTable 1 in the Supplement). The clusters' altitudes ranged from −372 to 5951 m above sea level.

### Statistical Analysis

To examine HAZ age trajectories in low- and middle-income countries,^19^ we used local polynomial regressions that regressed the child’s HAZ on their age in months. We ran separate regressions for children residing in clusters lower than 1500 m above sea level (n = 857 858) and children residing in clusters 1500 m or more above sea level (n = 106 441). We compared the regression lines and their 95% CIs between the 2 groups to assess whether the differences in growth trajectories were statistically different from zero. The weights in these regressions were based on the Epanechnikov kernel-density function, and the bandwidth was selected using the rule-of-thumb method.^20^

The HAZ of children was then regressed on the altitude of the DHS cluster in which the child resided using a local polynomial regression, where the weights and the bandwidth were selected using the methods described above. Unadjusted and adjusted linear regression models were used to quantify the HAZ deficit per increments of 1000 m above sea level in the altitude of the DHS cluster in which the child resided (eFigure 1 in the Supplement). Adjusted models controlled for differences in biological and underlying causes of linear growth faltering^4,19,21^: child age (set of binary variables for each month) and sex, maternal age and level of education (years), household wealth (access to electricity and ownership of radio, television, refrigerator, bicycle, motorcycle, car, and improved floor material), and binary variables capturing access to improved water and sanitation (eTable 2 in the Supplement). We also controlled for residence in a rural area and used subnational region (highest administrative unit in each country) fixed effects to control for economic, political, climatic, and other factors shared by the residents in the same administrative area. We had 1348 survey-specific subnational regions in the full sample. Both unadjusted and adjusted regressions were based on the full sample of children aged 0 to 59 months as well as subsamples consisting of different age groups: 0 to 5, 6 to 11, 12 to 23, and 24 to 59 months. In adjusted models, children with missing control variable values were omitted from the sample. We clustered our SEs at the subnational region level.^22^

We used multivariable regression models to assess how altitude was associated with immediate causes of undernutrition: inadequate dietary intake and disease.^4^ As measures of inadequate dietary intake, we used prevalence of exclusive breastfeeding (children aged 0-6 months), dietary diversity (age 6-23 months), and minimum acceptable diet (age 6-23 months) as dependent variables. For disease risk, we used incidence of diarrhea, fever, or cough in the 2 weeks preceding the survey (age 0-59 months). More details are provided in eAppendix 1 and eTable 3 in the Supplement. The adjusted logistic regressions were based on the same set of control variables as described above and the SEs were clustered at the subnational region level. The estimated HAZ deficits per 1000-m above sea level increments in altitude are expressed as odds ratios (ORs).

In the final part of the analysis, we restricted the sample to children residing in ideal home environments. Using the Karra et al guidelines,^8^ children were classified as having lived in an ideal home environment based on the following criteria: (1) singleton birth; (2) access to safe water and sanitation; (3) living in a house with finished floors, parents owned a television and a car; (4) born to highly educated mothers (>13 years of schooling); (5) born in a hospital; and (6) received BCG and first diphtheria, pertussis, and tetanus vaccinations (eAppendix 2 in the Supplement). Unadjusted and adjusted linear models were used to regress the HAZ of children living in ideal environments on the altitude of the cluster in which the child resided. Adjusted models controlled for child sex and age. We used heteroskedasticity robust SEs with this ideal home environment sample.

We ran multiple robustness checks to assess the sensitivity of our results. We assessed whether the multivariable regression results were robust to replacing the continuous altitude variable with a binary variable capturing 1500-m above sea level clusters. Climatic patterns differ in low- and high-altitude locations and may also directly affect linear growth. While our subnational fixed effects controlled for climatic differences between regions, we augmented the model with data on long-term average rainfall and temperature in the cluster. We explored sensitivity by adding maternal height as a control in the multivariable regression model. We restricted the sample to children whose mother had lived in the same cluster at least since conception. There were 18 countries that had no clusters in areas 1500 m or more above sea level. We assessed sensitivity by omitting these 18 countries from the sample. We explored sensitivity of the regression results by omitting each country from the sample. We assessed the association between altitude and stunting (HAZ <−2) using the same regression methods as described above. Stata, version 16.1 (StataCorp LLC) was used for all analyses.

## Results

In 2010, of the global population of 6907 million people, 842 million (12%) lived in locations 1500 m or more above sea level, with two-thirds residing in Asia (287 million) and Africa (276 million). The country- and region-specific populations living 1500 m or more above sea level are reported in eFigure 2 and eTable 4 in the Supplement. Most children in the DHS sample resided in clusters with altitudes below 500 m above sea level, and 106 441 children (11% of the full sample) resided in areas 1500 m or more above sea level (eTable 2 in the Supplement).

**Figure 1.**
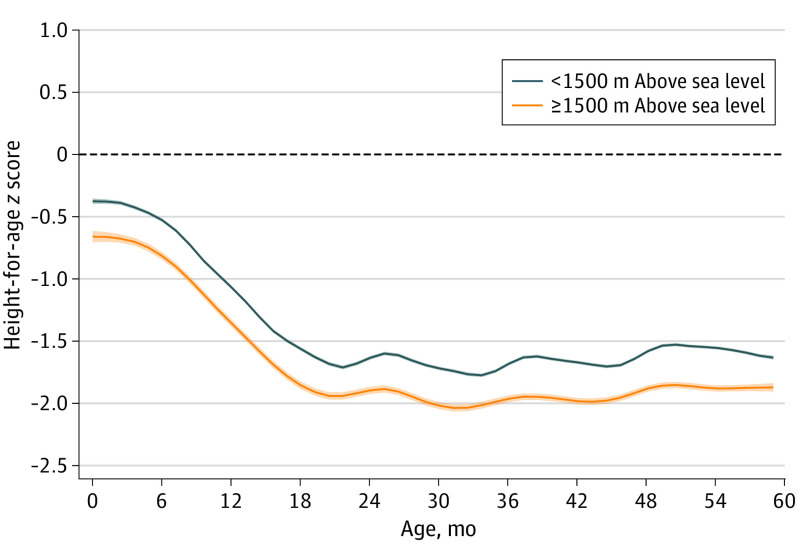
Association Between Residence at 1500 m or More Above Sea Level and Less Than 1500 m Above Sea Level and Length-for-Age/Height-for-Age *z* Score (HAZ) Age Trajectories Children residing at 1500 m or more above sea level (n = 106 441) grow, on average, on a lower trajectory compared with children in lower altitudes (n = 857 858) as well as the median child in the World Health Organization 2006 growth standard (the horizontal dashed line).^7^ The HAZ altitude deficit emerged at birth and remained throughout the 0- to 59-month study window. The local polynomial regressions were calculated using the Epanechnikov kernel-density function for the weights and the rule-of-thumb method to select the bandwidth. Shaded areas represent 95% CIs.

Local polynomial regressions between age and HAZ indicated that growth faltering for both altitude groups occurred during the first 24 months (Figure 1). Children residing 1500 m or more above sea level were, on average, born at a shorter length than children residing lower than 1500 m above sea level. After birth, the growth curve for children in areas 1500 m or more above sea level was consistently lower, implying limited catch-up to growth levels of children residing in areas lower than 1500 m above sea level.

**Figure 2.**
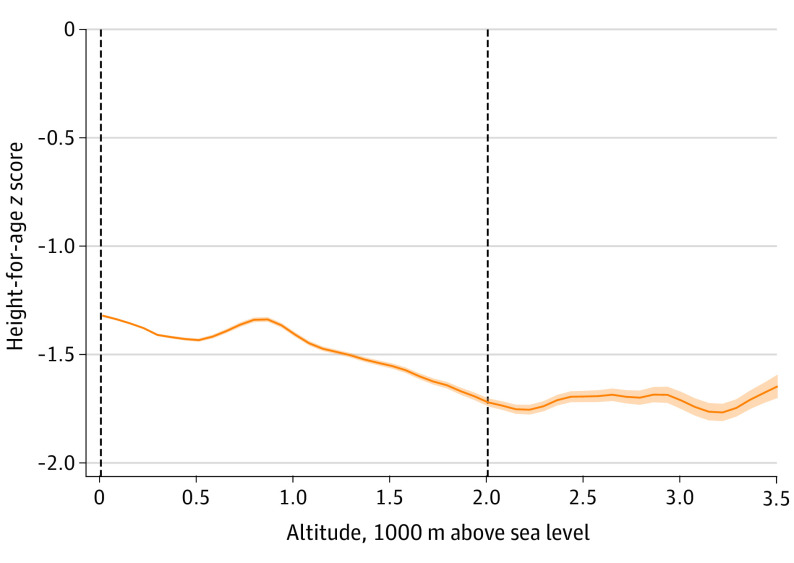
Association Between Altitude and Length-for-Age/Height-for-Age z Score (HAZ) The HAZ was negatively associated with altitude. This association was approximately linear through most part of the altitude distribution, indicating no clear altitude threshold for a rapid decrease in the HAZ. A local polynomial regression was calculated using the Epanechnikov kernel-density function for the weights and the rule-of-thumb method to select the bandwidth. Shaded area represents 95% CI. Dashed vertical lines represent the bottom and top 5% of the altitude distribution. Bottom and top 1% of the altitude distribution have been omitted; n = 944 980 children aged 0 to 59 months.

Local polynomial regressions showed that altitude was negatively associated with HAZ (Figure 2). This association was approximately linear until about the 95th percentile (approximately 2000 m above sea level) of the altitude distribution (Figure 2). Based on multivariable linear regressions adjusting for common risk factors for linear growth faltering (Table), a 1000-m above sea level increase in altitude was associated with a 0.163-unit (95% CI, −0.205 to −0.120 units) decrease in HAZ. The estimated HAZ altitude deficit was slightly larger for children aged 0 to 23 months compared with those aged 24 to 59 months.

**Table.** Regression Results for the Association Between Altitude and HAZ

Characteristic	Regression coefficient (95% CI)^a^	
	Unadjusted	Adjusted^b^
**All children**		
Age 0-59 mo		
No.	964 299	850 681
Altitude, 1000 m above sea level	−0.140 (−0.189 to −0.090)	−0.163 (−0.205 to −0.120)
Age 0-5 mo		
No.	98 472	86 680
Altitude, 1000 m above sea level	−0.111 (−0.155 to −0.067)	−0.184 (−0.239 to −0.128)
Age 6-11 mo		
No.	105 197	93 354
Altitude, 1000 m above sea level	−0.147 (−0.200 to −0.095)	−0.212 (−0.262 to −0.163)
Age 12-23 mo		
No.	200 286	177 232
Altitude, 1000 m above sea level	−0.149 (−0.204 to −0.094)	−0.178 (−0.219 to −0.137)
Age 24-59 mo		
No.	560 344	493 415
Altitude, 1000 m above sea level	−0.140 (−0.193 to −0.086)	−0.146 (−0.196 to −0.095)
**Children living in ideal home environment**		
Age 0-59 mo		
No.	1718	1718
Altitude, 1000 m above sea level	−0.174 (−0.333 to −0.016)	−0.183 (−0.341 to −0.025)

Abbreviation: HAZ, height-for-age *z* score.

^a^ Data are regression coefficients from linear regression models that regressed child HAZ on altitude (continuous measure). Each coefficient measures the associated change in HAZ when altitude is increased by 1000 m above sea level.

^b^ Adjusted estimates for all children were based on adjusted regression that controlled for child age (set of binary variables for each age-in-month) and sex, maternal age and educational level, household wealth, and access to improved water and sanitation, binary variable capturing rural areas, and subnational region (highest administrative unit in each country) fixed effects. For children living in an ideal home environment, adjustments were for the child’s age and sex.

**Figure 3.**
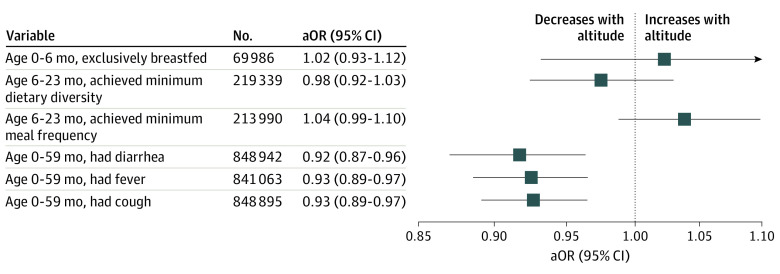
Adjusted Associations Between Altitude and Immediate Causes of Child Undernutrition Altitude was not associated with increased risk of inadequate diets and disease (ie, immediate causes of malnutrition); for some indicators (ie, diarrhea, fever, and cough risk) the opposite was true. Boxes are odds ratios (ORs) from 6 different logistic regressions that regressed each immediate cause of child undernutrition on altitude (1000 m above sea level). Bars are 95% CI. The ORs are based on an adjusted regression that controlled for child age (set of binary variables for each age [months]) and sex, maternal age and educational level, household wealth, and access to improved water and sanitation, binary variable capturing rural areas, and binary indicator variables capturing subnational regions (highest administrative unit in each country). ORs from unadjusted regressions are reported in eAppendix 5 in the Supplement. aOR indicates adjusted OR.

Adjusted multivariable logistic regressions indicated that increments in altitude were not associated with the likelihood that the child was exclusively breastfed, achieved minimum meal frequency, or achieved minimum dietary diversity (Figure 3). Altitude was negatively associated with the likelihood that the child had recently experienced diarrhea (adjusted OR [aOR], 0.92; 95% CI, 0.87-0.96), fever (aOR, 0.93; 95% CI, 0.89-0.97), or cough (aOR, 0.93; 95% CI, 0.89-0.97) (Figure 3). The unadjusted regression results are reported in eTable 5 in the Supplement.

**Figure 4.**
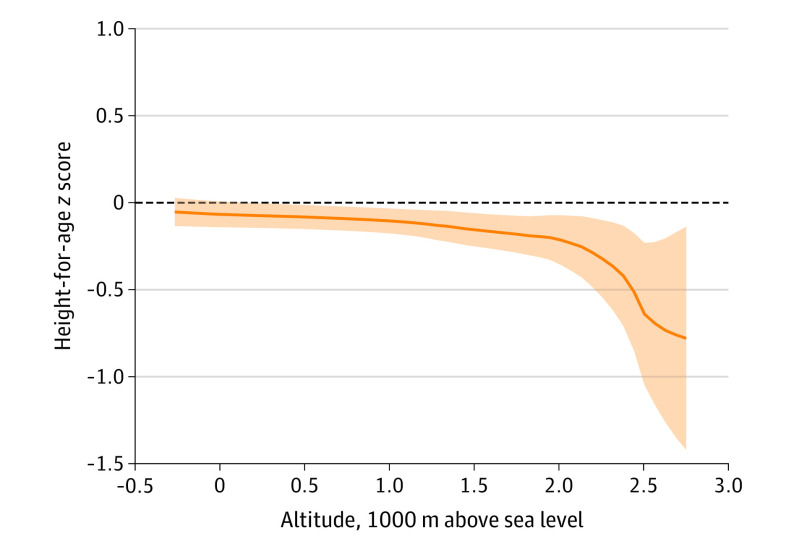
Association Between Altitude and Length-for Age/Height-for-Age *z* Score (HAZ) Among Children Residing in Ideal Home Environments Children residing in ideal home environments (n = 1718) grew, on average, at the same rate as the median child in the World Health Organization 2006 growth standard^7^ (horizontal dashed line), until the altitude reached approximately 500 m above sea level. A local polynomial regression was calculated using the Epanechnikov kernel-density function for the weights and the rule-of-thumb method to select the bandwidth. Shaded area represents 95% CI.

Of the 964 299 children in the sample, 1718 children were singletons and resided in ideal home environments (eAppendix 2 and eTable 6 in the Supplement). The HAZ distribution of children residing in ideal home environments was similar to the 2006 WHO HAZ distribution (eAppendix 2, eFigure 3, and eFigure 4 in the Supplement). However, a local polynomial regression showed that the average HAZ was approximately zero until 500 m above sea level, after which it began to decrease below the growth trajectory of the median child (HAZ, 0) in the 2006 WHO growth standard (Figure 4). Adjusting for child age and sex, a 1000-m above sea level increase in altitude was associated with a 0.183-unit (95% CI, −0.341 to −0.025 units) decrease in the HAZ among children residing in ideal home environments (Table).

Results from several robustness analyses are provided in the Supplement. The regression results were robust to measuring altitude using a binary variable capturing children residing 1500 m or more above sea level (eAppendix 3 and eTable 7 in the Supplement). Differences in the HAZ between low- and high-altitude populations were not due to differences in climate (eAppendix 4 and eTable 8 in the Supplement) or maternal stature (eAppendix 5 and eTable 9 in the Supplement). Restricting the sample to children whose mothers had lived in the same cluster at least since conception (eAppendix 6 and eTable 10 in the Supplement) or omitting countries that had no clusters at 1500 m or more above sea level (eAppendix 7 and eTable 11 in the Supplement) resulted in similar findings. The estimated altitude deficit in the full sample remained stable when individual countries were excluded from the sample (eAppendix 8 and eTable 12 in the Supplement). In addition, the estimated altitude deficit remained when using stunting (HAZ <−2) instead of the HAZ as the outcome variable (eAppendix 9, eFigure 5, and eTable 13 in the Supplement).

## Discussion

This study reports that children living 1500 m or more above sea level follow a lower growth trajectory than their peers living at lower altitudes. The negative association between altitude and the HAZ was approximately linear through most part of the altitude distribution, indicating no clear altitude threshold for an abrupt decrease in the HAZ. Those residing in ideal home environments grew at the same rate as the median child in the 2006 WHO growth standard until approximately 500 m above sea level. After 500 m above sea level, the average child HAZ significantly deviated from the growth curve of the median child in the 2006 WHO reference population. The estimated variation in linear growth based on altitude was stable from birth to 59 months, with no substantial catch-up. These estimated altitude-mediated growth deficits remained significant even after adjusting for differences in common risk factors for linear growth faltering. Adjusted regressions further indicated that altitude was not positively associated with the risk of inadequate diet and disease (ie, immediate causes of malnutrition); in some cases, the opposite was true.

The estimated altitude-mediated growth deficits are biologically meaningful. For example, for children living in Addis Ababa, Ethiopia (approximately 2355 m above sea level), the predicted altitude-mediated HAZ deficit is 0.4 SD, whereas for La Paz, Bolivia (approximately 3650 m above sea level), this deficit can be as high as 0.6 SD. Remedying this level of growth deficit in The Gambia (0.74 SD) required 4 decades of intensive nutrition-sensitive and nutrition-specific interventions.^23^ Given that close to 12% (842 million in 2010) of the world population, mostly in South and Central America, sub-Saharan Africa, and Asia, live at 1500 m or more above sea level and that these regions contribute the largest share of worldwide fetal-growth restriction and stunting, understanding the share of altitude-mediated growth deficits to this burden is important.

In this study, altitude-mediated growth deficits were apparent from birth and remained relatively stable over 6 to 59 months, suggesting that the association between altitude and growth is more pronounced during the perinatal period. Pregnancies at high altitudes are characterized by chronic hypoxia (inadequate oxygen supply) that is consistently associated with a higher risk of fetal growth restriction,^24,25^ which is a leading risk factor for linear growth faltering.^21,26^ While genetic adaptation to high altitude has been documented, this adaptation may take several generations and may not offer full protection against the growth faltering seen in children born at high altitudes.^16,27,28,29,30,31,32^ For example, women of high-altitude ancestry were able to partially cope with the hypoxic conditions through increased uterine artery blood flow during pregnancy, but such adaptations were not seen in short-term (150 years) high-altitude residents of European ancestors living in Colorado.^31^ Despite varying levels of genetic adaptations, both native and short-term high-altitude residents had lower average birth weights and lengths than lowland residents.^24,27,32^ To date, except for recommending that pregnant women not travel to high-altitude areas, there is little knowledge about interventions that can address the added risks of pregnancy complications at high altitudes.^33^

The association between residence at high altitude and linear growth has been a topic of interest since the 1950s. Lichty et al^34^ reported evidence that children in Lake City, Colorado (3048-3353 m above sea level), were born smaller than children from lower-altitude counties of Colorado. Studies from high-altitude sites in Colorado, South America, and Tibet also reported lower birth size^14,15,24,35^ and stature in infants and young children.^36,37,38^ However, subsequent studies argued that the observed growth deficits were due to harsher living conditions and poorer diets at higher altitudes.^13,17^ We have contributed to this literature by using a larger sample of children from 59 low- and middle-income countries, allowing us to consider the near full-spectrum of altitudes in which humans reside.

### Limitations

While gathering experimental evidence on this topic is unfeasible, we attempted to account for the role of confounding factors by using multivariable regression, assessing the association between altitude and immediate causes of undernutrition, and restricting the sample to children residing in ideal home environments. Yet, we cannot guarantee that the estimated altitude growth deficit is not explained, at least partly, by an unobservable confounding factor. Another limitation of our study is that, despite using data from multiple countries, the number of children that fulfilled the criteria for ideal home environment at higher altitudes was relatively small.

## Conclusions

Do the findings of this study indicate that the 2006 WHO growth standards should be adjusted downward for children residing 1500 m or more above sea level? Such adjustments would imply that altitude-mediated growth deficits are just physiologic adaptations and are not linked to more serious functional deficits. While this might be the case for a small proportion of the population that has resided in high-altitude areas over multiple generations to benefit from genetic adaptation,^39^ for most of the population, the growth impairments imply more serious functional deficits related to intrauterine growth restriction and impaired cognitive development.^40,41,42^ In addition, long before genetic adaptations take effect, hypoxia-induced fetal growth restriction can cause epigenetic changes that can persist throughout life, trigger growth faltering in subsequent generations, and contribute to intergenerational transmission of poor development.^43,44,45,46^ Instead of adjustments to growth standards, our findings suggest that attention and health care guidance for managing pregnancies in high-altitude settings are needed. A first step in this process is to unravel the complex relationship linking altitude, hypoxia, and fetal growth to identify useful interventions. Failing to address altitude-mediated growth deficits may result in a significant proportion of the world population not meeting the Sustainable Development Goals and World Health Assembly nutrition targets.

## Notes

Supplement. eTable 1. Demographic and Health Survey Data eTable 2. Summary Statistics, Full Sample eFigure 1. Distribution of the Demographic Health Survey (DHS) Sample by Altitude eAppendix 1. Immediate Causes of Undernutrition: Inadequate Dietary Intake and Disease eTable 3. Measures of Inadequate Dietary Indicators **eFigure 2.** The Number of People (in Millions) Residing Above 1,500 masl **eTable 4.** Number of People (in Millions) Residing in ≥1,500 masl in 2010, by Region **eTable 5.** Associations Between Altitude and Immediate Causes of Child Undernutrition **eAppendix 2. **Ideal Home Environments **eTable 6.** Number of Children Residing in Ideal Home Environment, by Country **eFigure 3.** Kernel-Density Plot of the HAZ Distribution of Children Residing in the Ideal Home Environment Sample Compared With the WHO-2006 HAZ (Standard Normal) Distribution **eFigure 4.** Distribution of the 'Ideal Home Environment' Sample by Altitude **eAppendix 3. **Robustness to Using Binary Altitude Variable **eTable 7.** Replicating Table 1 Using a Binary Altitude Variable That Obtains Value 1 if the Cluster Is Above 1,500 Meters and Zero Otherwise **eAppendix 4. **Robustness to Controlling for Climate Factors **eTable 8.** Regression Results for the (Unadjusted and Adjusted) Association Between Altitude and HAZ, After Controlling for Differences in Climatic Factors (Temperature and Rainfall) **eAppendix 5.** Robustness to Controlling for Maternal Stature **eTable 9.** Regression Results for the (Unadjusted and Adjusted) Association Between Altitude and HAZ, After Controlling for Differences in Maternal Height **eAppendix 6.** Robustness to Restricting the Sample to Children Whose Mother Had Lived in the Same Location at Least Since the Conception **eTable 10.** Regression Results for the (Unadjusted and Adjusted) Association Between Altitude and HAZ, After Restricting the Sample to Children Who Have Lived in the Same Location at Least Since the Conception **eAppendix 7. **Robustness to Omitting Low Altitude Countries From the Sample **eTable 11.** Regression Results for the (Unadjusted and Adjusted) Association Between Altitude and HAZ, After Restricting the Sample to Countries That Have Clusters Above 1,500 masl **eAppendix 8 .** Robustness to Excluding Each Country Individually From the Sample **eTable 12.** Regression Results for the Adjusted Association Between Altitude and HAZ, Excluding Each Country Individually From the Sample **eAppendix 9.** Robustness to Using Stunting as the Outcome Variable **eFigure 5.** Stunting-Age Trajectories in <1,500 masl and ≥1,500 masl (N = 964,299) **eTable 13.** Regression Results for the (Unadjusted and Adjusted) Association Between Altitude and Stunting (HAZ<-2) eReferences
