# Supplementary material for: Evaluation of Linear Growth at Higher Altitudes
Source: JAMA Pediatr. 2020 Aug 24;174(10):1–8. doi: 10.1001/jamapediatrics.2020.2386 (PMC7445632; doi:10.1001/jamapediatrics.2020.2386)
Supplement: Supplement. — eTable 1. Demographic and Health Survey Data eTable 2. Summary Statistics, Full Sample eFigure 1. Distribution of the Demographic Health Survey (DHS) Sample by Altitude eAppendix 1. Immediate Causes of Undernutrition: Inadequate Dietary Intake and Disease eTable 3. Measures of Inadequate Dietary Indicators eFigure 2. The Number of People (in Millions) Residing Above 1,500 masl eTable 4. Number of People (in Millions) Residing in ≥1,500 masl in 2010, by Region eTable 5. Associations Between Altitude and Immediate Causes of Child Undernutrition eAppendix 2. Ideal Home Environments eTable 6. Number of Children Residing in Ideal Home Environment, by Country eFigure 3. Kernel-Density Plot of the HAZ Distribution of Children Residing in the Ideal Home Environment Sample Compared With the WHO-2006 HAZ (Standard Normal) Distribution eFigure 4. Distribution of the 'Ideal Home Environment' Sample by Altitude eAppendix 3. Robustness to Using Binary Altitude Variable eTable 7. Replicating Table 1 Using a Binary Altitude Variable That Obtains Value 1 if the Cluster Is Above 1,500 Meters and Zero Otherwise eAppendix 4. Robustness to Controlling for Climate Factors eTable 8. Regression Results for the (Unadjusted and Adjusted) Association Between Altitude and HAZ, After Controlling for Differences in Climatic Factors (Temperature and Rainfall) eAppendix 5. Robustness to Controlling for Maternal Stature eTable 9. Regression Results for the (Unadjusted and Adjusted) Association Between Altitude and HAZ, After Controlling for Differences in Maternal Height eAppendix 6. Robustness to Restricting the Sample to Children Whose Mother Had Lived in the Same Location at Least Since the Conception eTable 10. Regression Results for the (Unadjusted and Adjusted) Association Between Altitude and HAZ, After Restricting the Sample to Children Who Have Lived in the Same Location at Least Since the Conception eAppendix 7. Robustness to Omitting Low Altitude Countries From the Sample eTable 11. Regr [file jamapediatr-e202386-s001.pdf]

**Supplementary Online Content**

Baye K, Hirvonen K. Evaluation of linear growth at higher altitudes. *JAMA Pediatr*. Published online August 24, 2020. doi:10.1001/jamapediatrics.2020.2386

**eTable 1.** Demographic and Health Survey Data
**eTable 2.** Summary Statistics, Full Sample
**eFigure 1.** Distribution of the Demographic Health Survey (DHS) Sample by Altitude
**eAppendix 1.** Immediate Causes of Undernutrition: Inadequate Dietary Intake and Disease
**eTable 3.** Measures of Inadequate Dietary Indicators
**eFigure 2.** The Number of People (in Millions) Residing Above 1,500 masl
**eTable 4.** Number of People (in Millions) Residing in ≥1,500 masl in 2010, by Region
**eTable 5.** Associations Between Altitude and Immediate Causes of Child Undernutrition
**eAppendix 2.** Ideal Home Environments
**eTable 6.** Number of Children Residing in Ideal Home Environment, by Country
**eFigure 3.** Kernel-Density Plot of the HAZ Distribution of Children Residing in the Ideal Home Environment Sample Compared With the WHO-2006 HAZ (Standard Normal) Distribution
**eFigure 4.** Distribution of the 'Ideal Home Environment' Sample by Altitude
**eAppendix 3.** Robustness to Using Binary Altitude Variable
**eTable 7.** Replicating Table 1 Using a Binary Altitude Variable That Obtains Value 1 if the Cluster Is Above 1,500 Meters and Zero Otherwise
**eAppendix 4.** Robustness to Controlling for Climate Factors
**eTable 8.** Regression Results for the (Unadjusted and Adjusted) Association Between Altitude and HAZ, After Controlling for Differences in Climatic Factors (Temperature and Rainfall)
**eAppendix 5.** Robustness to Controlling for Maternal Stature
**eTable 9.** Regression Results for the (Unadjusted and Adjusted) Association Between Altitude and HAZ, After Controlling for Differences in Maternal Height
**eAppendix 6.** Robustness to Restricting the Sample to Children Whose Mother Had Lived in the Same Location at Least Since the Conception
**eTable 10.** Regression Results for the (Unadjusted and Adjusted) Association Between Altitude and HAZ, After Restricting the Sample to Children Who Have Lived in the Same Location at Least Since the Conception
**eAppendix 7**. Robustness to Omitting Low Altitude Countries From the Sample
**eTable 11.** Regression Results for the (Unadjusted and Adjusted) Association Between Altitude and HAZ, After Restricting the Sample to Countries That Have Clusters Above 1,500 masl
**eAppendix 8 .** Robustness to Excluding Each Country Individually From the Sample
**eTable 12.** Regression Results for the Adjusted Association Between Altitude and HAZ, Excluding Each Country Individually From the Sample
**eAppendix 9.** Robustness to Using Stunting as the Outcome Variable
**eFigure 5.** Stunting-Age Trajectories in <1,500 masl and ≥1,500 masl (N = 964,299)
**eTable 13.** Regression Results for the (Unadjusted and Adjusted) Association Between Altitude and Stunting (HAZ<-2)
**eReferences**

This supplementary material has been provided by the authors to give readers additional information about their work.

© 2020 American Medical Association. All rights reserved. 

eTable 1: Demographic and Health Survey data: List of countries used in the analysis, sample sizes and altitude ranges by country[a]

| | Timing of the survey: | | Sample size | Altitude: | | |
|---|---|---|---|---|---|---|
| Country | First interview year | Last interview year | | mean | minimum | maximum |
| Albania | 2008 | 2009 | 1,452 | 432 | -2 | 1,606 |
| Albania | 2017 | 2018 | 2,446 | 293 | -3 | 1,667 |
| Angola | 2015 | 2016 | 6,359 | 861 | 4 | 2,068 |
| Armenia | 2015 | 2016 | 1,581 | 1,286 | 537 | 2,331 |
| Azerbaijan | 2006 | 2006 | 3,815 | 281 | -27 | 1,850 |
| Bangladesh | 1999 | 2000 | 5,427 | 18 | 2 | 247 |
| Bangladesh | 2004 | 2004 | 5,978 | 15 | 2 | 167 |
| Bangladesh | 2007 | 2007 | 5,349 | 14 | 2 | 137 |
| Bangladesh | 2011 | 2011 | 7,740 | 16 | 1 | 247 |
| Benin | 1996 | 1996 | 2,609 | 181 | 1 | 510 |
| Benin | 2001 | 2001 | 4,463 | 159 | 1 | 540 |
| Benin | 2011 | 2012 | 10,110 | 157 | 1 | 622 |
| Bolivia | 2008 | 2008 | 7,751 | 2,240 | 104 | 4,740 |
| Burkina Faso | 1993 | 1993 | 4,497 | 314 | 215 | 551 |
| Burkina Faso | 1998 | 1999 | 4,582 | 306 | 221 | 521 |
| Burkina Faso | 2003 | 2003 | 8,467 | 309 | 189 | 524 |
| Burkina Faso | 2010 | 2010 | 6,259 | 308 | 203 | 559 |
| Burundi | 2010 | 2010 | 3,469 | 1,492 | 716 | 2,271 |
| Burundi | 2016 | 2017 | 6,019 | 1,511 | 775 | 2,572 |
| Cambodia | 2000 | 2000 | 3,669 | 45 | 1 | 733 |
| Cambodia | 2005 | 2005 | 3,569 | 51 | 3 | 733 |
| Cambodia | 2010 | 2010 | 3,726 | 52 | 3 | 733 |
| Cambodia | 2014 | 2014 | 4,317 | 49 | -9 | 752 |
| Cameroon | 2004 | 2004 | 3,250 | 676 | 13 | 2,011 |
| Cameroon | 2011 | 2011 | 5,113 | 669 | 12 | 2,351 |
| Central African Republic | 1994 | 1995 | 2,389 | 521 | 357 | 1,063 |
| Chad | 2014 | 2015 | 20,016 | 420 | 217 | 1,712 |
| Colombia | 2010 | 2010 | 15,714 | 772 | -1 | 3,533 |
| Comoros | 2012 | 2012 | 2,439 | 428 | 12 | 1,610 |
| Congo Democratic Republic | 2007 | 2007 | 3,312 | 760 | 14 | 2,482 |
| Congo Democratic Republic | 2013 | 2014 | 8,195 | 700 | 12 | 2,422 |
| Cote d'Ivoire | 1994 | 1994 | 3,471 | 208 | 3 | 672 |
| Cote d'Ivoire | 1998 | 1999 | 1,569 | 185 | 3 | 612 |
| Cote d'Ivoire | 2011 | 2012 | 3,146 | 245 | 2 | 669 |
| Dominican Republic | 2007 | 2007 | 9,393 | 206 | -37 | 1,598 |
| Dominican Republic | 2013 | 2013 | 3,224 | 178 | -12 | 1,538 |
| Egypt | 1992 | 1992 | 7,589 | 33 | -43 | 273 |
| Egypt | 1995 | 1995 | 10,632 | 45 | -41 | 444 |

© 2020 American Medical Association. All rights reserved. 

| | | | | | | |
|---|---|---|---|---|---|---|
| Egypt | 2000 | 2000 | 10,553 | 42 | -24 | 1,885 |
| Egypt | 2003 | 2003 | 5,346 | 36 | -41 | 302 |
| Egypt | 2005 | 2005 | 12,218 | 42 | -41 | 881 |
| Egypt | 2008 | 2008 | 9,839 | 39 | -49 | 1,885 |
| Ethiopia | 2000 | 2000 | 8,824 | 1,806 | 185 | 3,425 |
| Ethiopia | 2005 | 2005 | 3,913 | 1,797 | 185 | 3,470 |
| Ethiopia | 2011 | 2011 | 9,397 | 1,628 | -52 | 3,417 |
| Ethiopia | 2016 | 2016 | 8,855 | 1,584 | 180 | 3,455 |
| Gabon | 2012 | 2012 | 3,396 | 260 | 6 | 770 |
| Ghana | 1993 | 1993 | 1,931 | 172 | -3 | 530 |
| Ghana | 1998 | 1998 | 2,796 | 180 | 0 | 601 |
| Ghana | 2003 | 2003 | 3,139 | 183 | -3 | 530 |
| Ghana | 2008 | 2008 | 2,394 | 178 | 1 | 464 |
| Ghana | 2014 | 2014 | 2,670 | 172 | 1 | 556 |
| Guatemala | 2014 | 2015 | 11,744 | 1,208 | 5 | 3,331 |
| Guinea | 1999 | 1999 | 4,504 | 387 | 3 | 1,172 |
| Guinea | 2005 | 2005 | 2,651 | 441 | 3 | 1,131 |
| Guinea | 2012 | 2012 | 3,175 | 441 | 3 | 1,279 |
| Guyana | 2009 | 2009 | 1,562 | 85 | 1 | 925 |
| Haiti | 2000 | 2000 | 5,568 | 329 | 7 | 1,569 |
| Haiti | 2005 | 2006 | 2,499 | 319 | 4 | 1,788 |
| Haiti | 2012 | 2012 | 3,972 | 336 | 7 | 1,569 |
| Haiti | 2016 | 2017 | 5,613 | 293 | 2 | 1,607 |
| Honduras | 2011 | 2012 | 9,824 | 672 | 4 | 1,941 |
| India | 2015 | 2016 | 231,951 | 371 | -4 | 5,951 |
| Jordan | 2002 | 2002 | 4,884 | 729 | -372 | 1,560 |
| Jordan | 2007 | 2007 | 4,570 | 752 | -372 | 1,560 |
| Jordan | 2012 | 2012 | 6,309 | 745 | -372 | 1,560 |
| Kenya | 2003 | 2003 | 4,779 | 1,345 | 1 | 2,929 |
| Kenya | 2008 | 2009 | 5,191 | 1,218 | 3 | 3,560 |
| Kyrgyz Republic | 2012 | 2012 | 4,010 | 1,307 | 540 | 3,401 |
| Lesotho | 2004 | 2004 | 1,327 | 1,998 | 1,458 | 2,821 |
| Lesotho | 2009 | 2010 | 1,622 | 2,010 | 1,491 | 3,059 |
| Lesotho | 2014 | 2014 | 1,312 | 1,910 | 1,452 | 2,815 |
| Liberia | 2007 | 2007 | 4,374 | 137 | 6 | 682 |
| Liberia | 2013 | 2013 | 3,214 | 158 | 6 | 571 |
| Madagascar | 1997 | 1997 | 3,052 | 620 | 2 | 2,028 |
| Madagascar | 2008 | 2009 | 5,148 | 618 | 2 | 1,970 |
| Malawi | 2000 | 2000 | 9,411 | 841 | 49 | 1,833 |
| Malawi | 2004 | 2004 | 8,312 | 851 | 39 | 1,833 |
| Malawi | 2010 | 2010 | 4,612 | 839 | 36 | 1,795 |
| Malawi | 2015 | 2016 | 5,149 | 820 | 43 | 1,762 |
| Mali | 1995 | 1996 | 4,292 | 313 | 44 | 542 |
| Mali | 2006 | 2006 | 11,312 | 327 | 31 | 761 |
| Mali | 2012 | 2013 | 4,450 | 318 | 39 | 604 |
| Moldova | 2005 | 2005 | 1,340 | 131 | 20 | 266 |
| Morocco | 2003 | 2004 | 5,570 | 512 | 1 | 2,635 |

© 2020 American Medical Association. All rights reserved. 

| | | | | | | |
|---|---|---|---|---|---|---|
| Mozambique | 2011 | 2011 | 9,505 | 314 | 4 | 1,544 |
| Myanmar | 2015 | 2016 | 4,177 | 291 | -7 | 1,800 |
| Namibia | 2000 | 2000 | 2,998 | 1,132 | 4 | 1,783 |
| Namibia | 2006 | 2007 | 3,686 | 1,151 | 4 | 1,965 |
| Namibia | 2013 | 2013 | 1,837 | 1,107 | 4 | 1,921 |
| Nepal | 2001 | 2001 | 6,216 | 825 | 64 | 4,416 |
| Nepal | 2006 | 2006 | 5,268 | 876 | 64 | 4,864 |
| Nepal | 2011 | 2011 | 2,346 | 907 | 65 | 3,203 |
| Nepal | 2016 | 2016 | 2,372 | 713 | 68 | 3,110 |
| Niger | 1998 | 1998 | 3,910 | 328 | 172 | 703 |
| Nigeria | 1990 | 1990 | 5,942 | 283 | 4 | 1,411 |
| Nigeria | 2003 | 2003 | 4,527 | 338 | 4 | 1,264 |
| Nigeria | 2008 | 2008 | 20,590 | 333 | 1 | 3,884 |
| Nigeria | 2013 | 2013 | 25,269 | 312 | 3 | 1,552 |
| Pakistan | 2017 | 2018 | 4,139 | 726 | 2 | 5,209 |
| Peru | 2000 | 2000 | 11,666 | 1,801 | 1 | 4,814 |
| Peru | 2003 | 2008 | 10,471 | 1,540 | 1 | 4,814 |
| Peru | 2009 | 2009 | 9,391 | 1,633 | 1 | 4,679 |
| Rwanda | 2005 | 2005 | 3,664 | 1,732 | 1,045 | 2,522 |
| Rwanda | 2010 | 2010 | 4,104 | 1,719 | 1,045 | 3,193 |
| Rwanda | 2014 | 2015 | 3,563 | 1,705 | 951 | 2,768 |
| Senegal | 1992 | 1993 | 4,598 | 29 | 3 | 257 |
| Senegal | 2005 | 2005 | 2,805 | 31 | 2 | 256 |
| Senegal | 2010 | 2011 | 3,723 | 33 | 3 | 257 |
| Senegal | 2012 | 2013 | 5,988 | 36 | 3 | 257 |
| Sierra Leone | 2008 | 2008 | 2,165 | 175 | 4 | 715 |
| Sierra Leone | 2013 | 2013 | 4,324 | 148 | 3 | 690 |
| South Africa | 2016 | 2016 | 1,113 | 994 | 13 | 1,764 |
| Swaziland | 2006 | 2007 | 2,029 | 683 | 111 | 1,429 |
| Tajikistan | 2012 | 2012 | 4,418 | 1,110 | 338 | 4,393 |
| Tajikistan | 2017 | 2017 | 5,881 | 906 | 298 | 3,875 |
| Tanzania | 1999 | 1999 | 2,511 | 790 | 13 | 2,148 |
| Tanzania | 2010 | 2010 | 6,629 | 897 | 4 | 2,509 |
| Tanzania | 2015 | 2016 | 9,001 | 961 | 6 | 2,602 |
| Timor-Leste | 2009 | 2010 | 7,810 | 537 | 3 | 1,995 |
| Timor-Leste | 2016 | 2016 | 5,909 | 473 | -1 | 2,179 |
| Togo | 1998 | 1998 | 3,711 | 228 | 2 | 767 |
| Uganda | 2000 | 2001 | 5,201 | 1,285 | 628 | 3,573 |
| Uganda | 2006 | 2006 | 2,392 | 1,214 | 626 | 2,406 |
| Uganda | 2011 | 2011 | 2,065 | 1,193 | 625 | 2,310 |
| Uganda | 2016 | 2016 | 4,423 | 1,199 | 615 | 2,357 |
| Yemen | 2013 | 2013 | 13,624 | 1,402 | 1 | 3,091 |
| Zambia | 2007 | 2007 | 5,220 | 1,161 | 422 | 1,698 |
| Zambia | 2013 | 2014 | 11,576 | 1,171 | 372 | 1,698 |
| Zimbabwe | 1999 | 1999 | 2,680 | 1,082 | 413 | 1,595 |
| Zimbabwe | 2005 | 2006 | 4,012 | 1,051 | 330 | 1,761 |
| Zimbabwe | 2010 | 2011 | 4,213 | 1,082 | 383 | 1,857 |

© 2020 American Medical Association. All rights reserved. 

| | | | | | | |
|---|---|---|---|---|---|---|
| Zimbabwe | 2015 | 2015 | 4,957 | 1,101 | 229 | 1,754 |

[a]All DHS surveys used in the analysis and shows the first and last interview year, the sample size (number of children under 5 for which height was recorded) and mean, minimum and maximum altitude.

© 2020 American Medical Association. All rights reserved. 

**eTable 2: Summary statistics, full sample**

| | N | mean | std. dev. | min | max |
|---|---|---|---|---|---|
| **Locational characteristics:** | | | | | |
| Altitude (in 1,000 masl) of the cluster | 964,299 | 0.60 | 0.72 | -0.37 | 5.95 |
| Rural area (0/1) | 964,299 | 0.70 | 0.46 | 0 | 1 |
| **Child's characteristics:** | | | | | |
| Height-for-age z-score | 964,299 | -1.41 | 1.74 | -6 | 6 |
| Age of child in months | 964,299 | 28.65 | 17.17 | 0 | 59 |
| Child is a boy (0/1) | 964,299 | 0.51 | 0.50 | 0 | 1 |
| **Mother's characteristics:** | | | | | |
| Mother's age in years | 964,299 | 28.43 | 6.49 | 13 | 49 |
| Mother's education in single years | 950,373 | 4.87 | 4.78 | 0 | 27 |
| **Water and sanitation:** | | | | | |
| Improved water (piped, tube well & bottled/sachet) (0/1) | 964,299 | 0.66 | 0.47 | 0 | 1 |
| Improved latrine (flush toilet) (0/1) | 964,299 | 0.29 | 0.45 | 0 | 1 |
| **Household wealth:** | | | | | |
| Has electricity (0/1) | 944,331 | 0.53 | 0.50 | 0 | 1 |
| Has radio (0/1) | 956,573 | 0.45 | 0.50 | 0 | 1 |
| Has TV (0/1) | 956,564 | 0.43 | 0.49 | 0 | 1 |
| Has refrigerator (0/1) | 920,687 | 0.23 | 0.42 | 0 | 1 |
| Has bicycle (0/1) | 940,054 | 0.34 | 0.47 | 0 | 1 |
| Has motorcycle (0/1) | 916,354 | 0.20 | 0.40 | 0 | 1 |
| Has car (0/1) | 912,985 | 0.07 | 0.26 | 0 | 1 |
| Has improved floor material (0/1) | 954,954 | 0.45 | 0.50 | 0 | 1 |

*Note: (0/1) = binary variable.*

© 2020 American Medical Association. All rights reserved. 

**eFigure 1: Distribution of the Demographic Health Survey (DHS) sample by altitude**

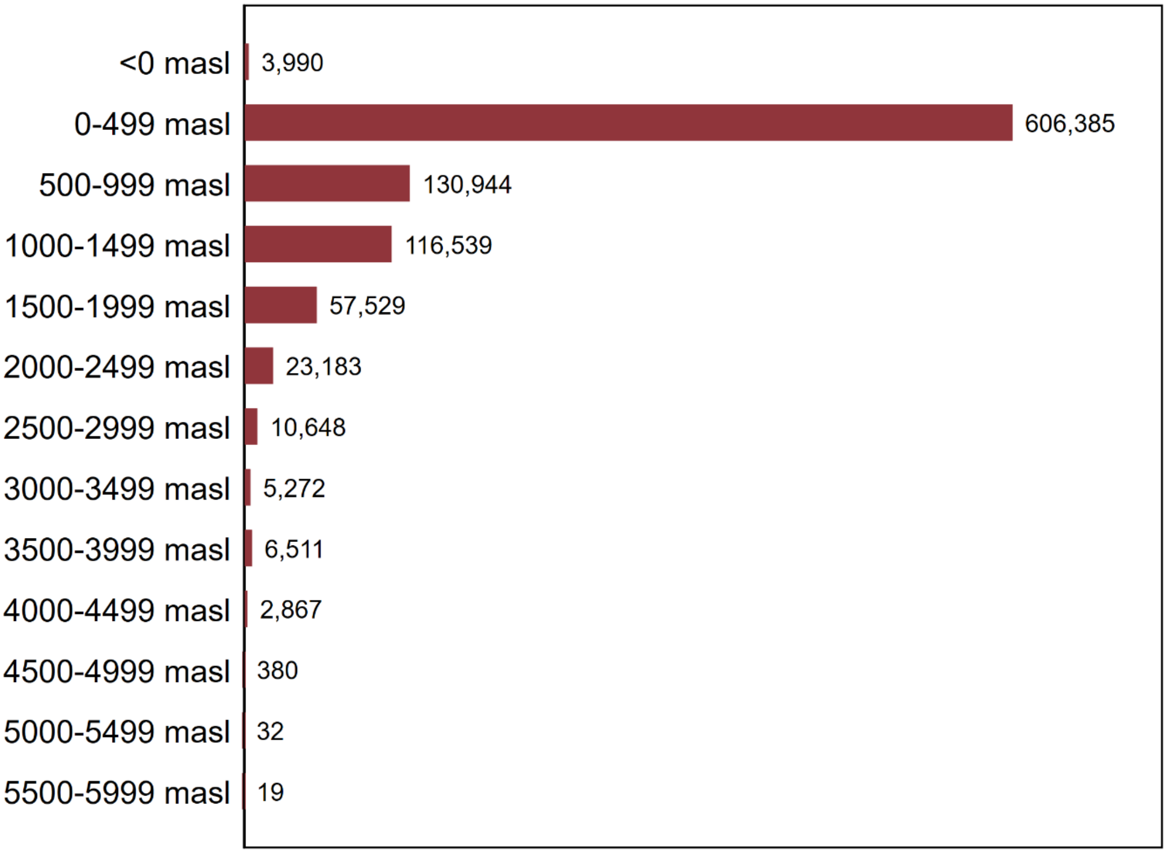


*Note: Data are number of children. Most children in the DHS sample reside below 500 masl while 106,441 children (11 % of the full sample) reside above 1,500 masl. N = 964,299*

© 2020 American Medical Association. All rights reserved. 

## Appendix 1: Immediate causes of undernutrition: inadequate dietary intake and disease

Inadequate dietary intake and disease are considered as the immediate causes of child undernutrition [1,2]. As measures of inadequate dietary intake, we used exclusive breastfeeding (0–6 month old children), dietary diversity (6–23 m), and minimum meal frequency diet (6–23 m) as dependent variables. These dietary indicators were calculated following WHO [3] guidelines: see eTable 3. Questions about child's dietary diversity were introduced in DHS phase–5 (roughly mid-2000s) and therefore the sample for this indicator is restricted to surveys that were based on phase 5 or a later questionnaire. Dietary diversity is based on caregiver's responses to yes/no questions about child's consumption of different food items in the last 24 hours. These food items are then grouped in to seven food groups based on their nutritional qualities. A child who consumed from all seven food groups received a score 7, and a child that consumed only from one food group receives a score 1, and so on. These indicators were developed and validated to proxy diet quality and energy intake in children [4,5] and have been consistently associated with child growth [6,7]. To date, they are the best, mutually comparable, indicators recommended by the WHO that are available for almost all LMIC with a DHS [8].

To measure disease risk, we used incidence of diarrhoea, fever or cough in the 2 weeks preceding the interview. DHS data collect these data for all children 0–59 m.

© 2020 American Medical Association. All rights reserved. 

## eTable 3: Measures of inadequate dietary indicators

| Indicator name | Definition |
|---|---|
| Exclusive breastfeeding under 6 months | Proportion of infants 0–5 months of age who are fed exclusively with breast milk. |
| Minimum dietary diversity | Proportion of children 6–23 months of age who receive foods from 4 or more food groups. |
| Minimum meal frequency | Proportion of breastfed and non-breastfed children 6–23 months of age who receive solid, semisolid, or soft foods (but also including milk feeds for non-breastfed children) the minimum number of times or more. |

Source: WHO [3].

© 2020 American Medical Association. All rights reserved. 

.

**eFigure 2: The number of people (in millions) residing above 1,500 masl**

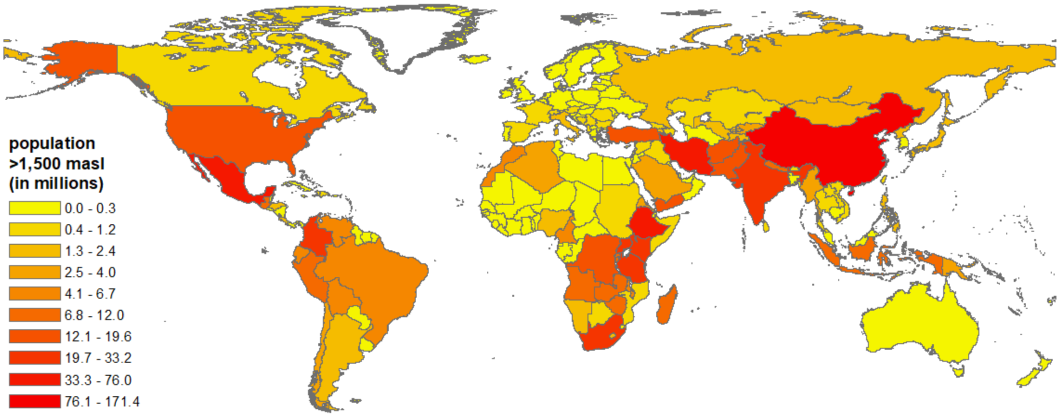


The map is based on Center for International Earth Science Information Network (CIESIN, Columbia University) data on population disaggregated by altitude. Countries with large populations residing above 1,500 masl are mainly located in Asia, sub-Saharan Africa and South and Central America

© 2020 American Medical Association. All rights reserved. 

**eTable 4: Number of people (in millions) residing in ≥1,500 masl in 2010, by region**

| | N | % |
|---|---|---|
| **World total** | **841.6** | **100%** |
| **Africa** | **287.5** | **34%** |
| Eastern Africa | 217.7 | 26% |
| Middle Africa | 29.8 | 3.5% |
| Northern Africa | 8.3 | 1.0% |
| Southern Africa | 29.4 | 3.5% |
| Western Africa | 2.2 | 0.3% |
| **Americas** | **164.6** | **20%** |
| Caribbean | 1.2 | 0.1% |
| Central America | 83.5 | 10% |
| Northern America | 15.2 | 1.8% |
| South America | 64.8 | 7.7% |
| **Asia** | **375.6** | **45%** |
| Central Asia | 7.7 | 0.9% |
| Eastern Asia | 177.9 | 21% |
| South-Eastern Asia | 15.7 | 1.9% |
| Southern Asia | 129.1 | 15% |
| Western Asia | 45.2 | 5.4% |
| **Europe** | **11.1** | **1.3%** |
| Eastern Europe | 3.1 | 0.4% |
| Northern Europe | 0.1 | 0.0% |
| Southern Europe | 5.4 | 0.6% |
| Western Europe | 2.5 | 0.3% |
| **Oceania** | **2.8** | **0.3%** |
| Australia + New Zealand | 0.1 | 0.0% |
| Melanesia | 2.7 | 0.3% |
| Micronesia | 0 | 0.0% |
| Polynesia | 0 | 0.0% |

High altitude populations are concentrated in East-Africa (26 %), Central and South America (18 %), and East and South Asia (36 %).

*Source: Center for International Earth Science Information Network - CIESIN - Columbia University* [9]

© 2020 American Medical Association. All rights reserved. 

## eTable 5: Associations between altitude and immediate causes of child undernutrition

| | | | | |
|---|---|---|---|---|
| | **Dependent variable: child was exclusively breastfed** | | | |
| | *unadjusted* | | *adjusted* | |
| age range: 0-6 mo | 1.295*** | [1.188,1.410] | 1.023 | [0.932,1.122] |
| | N = 78,234 | | N = 69,986 | |
| | **Dependent variable: child achieved minimum dietary diversity** | | | |
| | *unadjusted* | | *adjusted* | |
| age range: 6-23 mo | 1.259*** | [1.124,1.411] | 0.975 | [0.924,1.030] |
| | N = 234,547 | | N = 219,339 | |
| | **Dependent variable: child achieved minimum meal frequency** | | | |
| | *unadjusted* | | *adjusted* | |
| age range: 6-23 mo | 1.382*** | [1.179,1.619] | 1.041 | [0.988,1.098] |
| | N = 242,125 | | N = 213,990 | |
| | **Dependent variable: child had diarrhoea** | | | |
| | *unadjusted* | | *adjusted* | |
| age range: 0-59 mo | 1.132*** | [1.077,1.189] | 0.916*** | [0.870,0.964] |
| | N = 962,450 | | N = 848,942 | |
| | **Dependent variable: child had fever** | | | |
| | *unadjusted* | | *adjusted* | |
| age range: 0-59 mo | 1.025 | [0.964,1.090] | 0.925*** | [0.886,0.965] |
| | N = 954,544 | | N = 841,063 | |
| | **Dependent variable: child had cough** | | | |
| | *unadjusted* | | *adjusted* | |
| age range: 0-59 mo | 1.178*** | [1.101,1.261] | 0.927*** | [0.891,0.965] |
| | N = 962,381 | | N = 848,895 | |

Data are odds ratios from a logistic regression. The 95% confidence intervals (CI) are reported in brackets. Statistical significance denoted at * $p < 0.05$, ** $p < 0.01$, *** $p < 0.001$. The estimates in 'adjusted' column are based on adjusted regression that controls for child age (set of binary variables for each age-in-month) and sex, maternal age and education, household wealth and access to improved water and sanitation, binary variable capturing rural areas, and binary indicator variables capturing subnational regions (highest administrative unit in each country).

© 2020 American Medical Association. All rights reserved. 

## Appendix 2: Ideal Home Environments

To assess the validity of the WHO growth standards, Karra, Subramanian, Fink [10] used DHS data from 63 countries (169 surveys) to identify children living in environments in which they were more likely to achieve their full genetic growth potential. The authors used six criteria for an 'Ideal home environment':

1) Children were single births;
2) Access to safe water and sanitation;
3) Household lived in a house with finished floors, owned a television, and a car;
4) Children were born to highly educated mothers (more than 13 years of schooling);
5) Children were born in hospitals; and
6) Children had received Bacillus Calmette–Guérin, first diphtheria, pertussis, and tetanus vaccinations.

Out of 878,249 children less than 5 years of age with recorded height in DHS surveys between 1990 and 2014, Karra *et al* identified 1,006 children who lived in ideal home environments. The authors then compared the height-for-age Z-score distribution of this sample of 1,006 children to the WHO growth standard. Children living in ideal home environments had very similar HAZ scores than the WHO reference sample (see eFigure 3 below). The mean HAZ score in the WHO reference sample is, by construction, 0 with SD = 1. The mean HAZ score in the ideal home environment sample was estimated as 0.043, not statistically different from zero (95% CI: -0.039; 0.125).

We replicated this analysis using updated DHS data until 2017. We restricted the sample to surveys that recorded altitude from 128 surveys administered in 55 countries that recorded altitude of the cluster. Out of 921,665 children, we identified 1,718 children (0.19%) who resided in an 'ideal home environment' as defined by Karra *et al*. eTable 6 shows the geographical distribution of these children. The empirical HAZ distribution of the children residing in ideal home environments (green solid line) is similar to the distribution of the WHO reference population (standard normal by construction). Using linear regression and controlling for country-year fixed effects, the mean was estimated as -0.091(SD: 1.436, 95%-CI: -0.163; -0.020). This mean is statistically different from zero at 95-% level, and lower what was estimated by Karra *et al*. Figure 4 in the main text suggest that HAZ<0 is driven by children residing at higher altitudes.

Finally, eFigure 4 below shows that, in line with the full DHS sample (see eFigure 1 above), more than half of the children living in ideal home environments were found residing below 500 masl.

© 2020 American Medical Association. All rights reserved. 

**eTable 6: Number of children residing in ideal home environment, by country**

| **Country** | **N** | **%** |
|---|---|---|
| Albania | 34 | 1.98 |
| Colombia | 40 | 2.33 |
| Congo Democratic Republic | 5 | 0.29 |
| Dominican Republic | 356 | 20.72 |
| Egypt | 13 | 0.76 |
| Ethiopia | 1 | 0.06 |
| Ghana | 26 | 1.51 |
| Guyana | 5 | 0.29 |
| Haiti | 19 | 1.11 |
| Honduras | 112 | 6.52 |
| India | 149 | 8.67 |
| Jordan | 713 | 41.5 |
| Kenya | 14 | 0.81 |
| Moldova | 5 | 0.29 |
| Morocco | 6 | 0.35 |
| Mozambique | 1 | 0.06 |
| Namibia | 2 | 0.12 |
| Nigeria | 174 | 10.13 |
| Peru | 15 | 0.87 |
| Sierra Leone | 2 | 0.12 |
| Tanzania | 4 | 0.23 |
| Timor-Leste | 17 | 0.99 |
| Uganda | 3 | 0.17 |
| Zambia | 2 | 0.12 |
| **Total** | **1,718** | **100** |

© 2020 American Medical Association. All rights reserved. 

**eFigure 3: Kernel-density plot of the HAZ distribution of children residing in the ideal home environment sample compared with the WHO-2006 HAZ (standard normal) distribution**

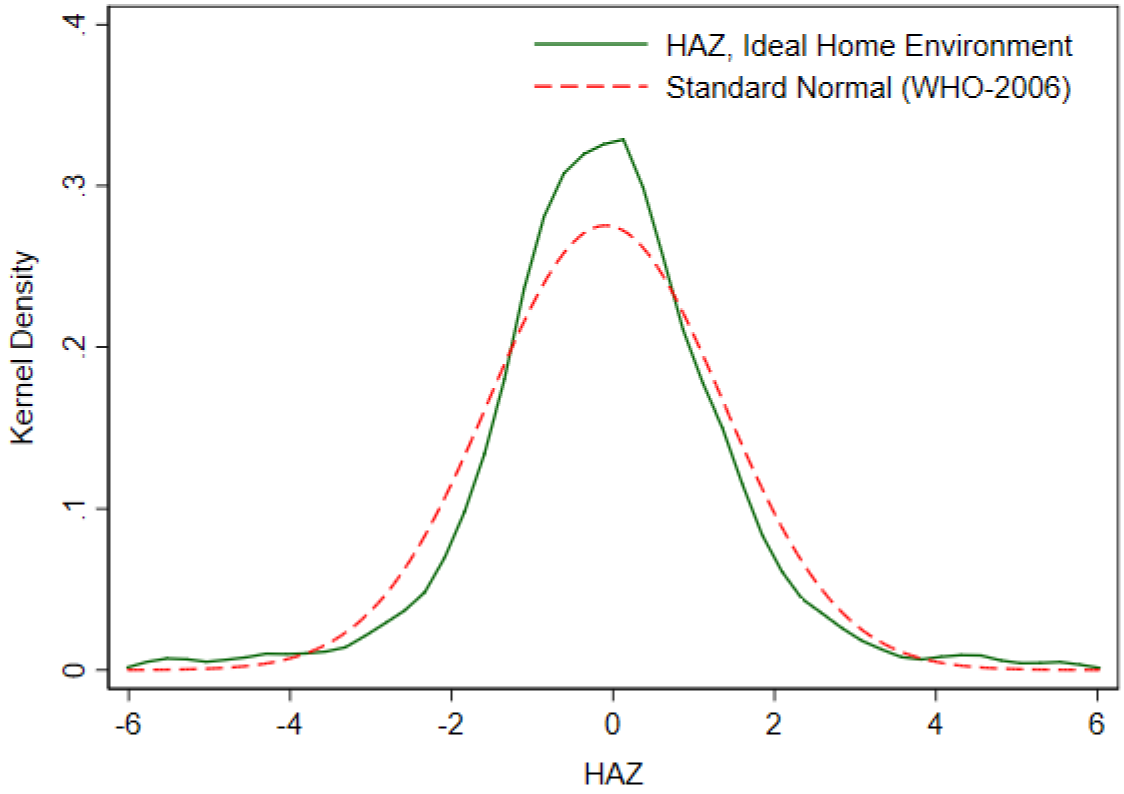


*Note: N = 1,718; mean HAZ: -0.091 (SD: 1.503, 95%-CI: -0.163; -0.020).*

© 2020 American Medical Association. All rights reserved. 

**eFigure 4: Distribution of the 'ideal home environment' sample by altitude**

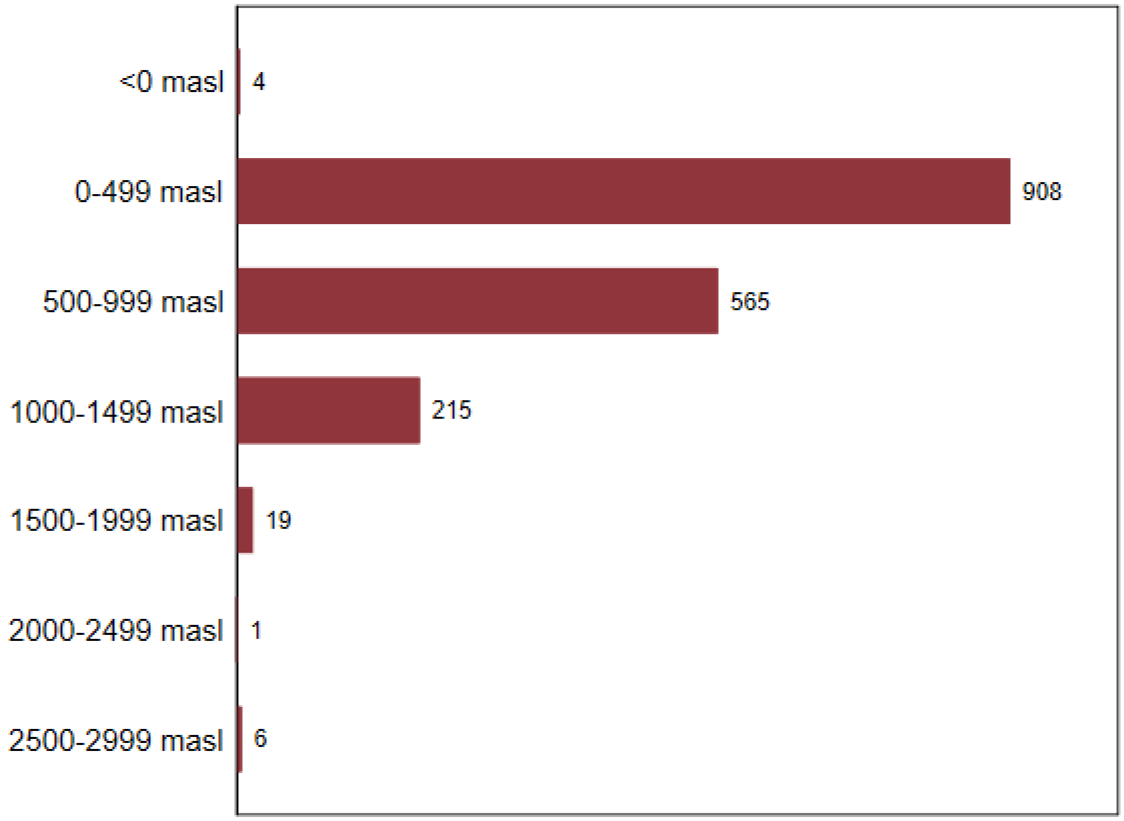


*Note: N = 1,718.*

© 2020 American Medical Association. All rights reserved. 

## eAppendix 3: Robustness to using binary altitude variable

In the main text, we used continuous altitude (in 1,000—masl) measure in our multivariable regression models (Table 1). This was motivated by the finding in Figure 2 according to which the relationship between altitude and HAZ was approximately linear through most part of the altitude distribution indicating no clear altitude threshold for rapid fall in HAZ. Consequently, the use of binary indicator would not be advised from a statistical point of view because it results in a loss of useful information and statistical power [11]. However, our regression results are robust to measuring altitude using a binary variable that obtains value 1 if the cluster in which the child is located is ≥1,500 meters and zero otherwise. According to the adjusted regression coefficient reported in eTable 7, high altitude (i.e., ≥1,500 masl) residence is associated with a 0.179 (95-% CI: -0.-0.235, to -0.123) unit lower child HAZ compared to low altitude residence (i.e., <1,500 masl), after adjusting for common risk factors.

© 2020 American Medical Association. All rights reserved. 

**eTable 7: Replicating Table 1 using a binary altitude variable that obtains value 1 if the cluster is above 1,500 meters and zero otherwise**

| | **age range: 0–59 months** | | | |
|---|---|---|---|---|
| | *unadjusted* | | *adjusted* | |
| above 1,500 altitude | -0.286*** | [-0.386,-0.186] | -0.179*** | [-0.235,-0.123] |
| | N=964,299 | | N=850,681 | |
| | **age range: 0–5 months** | | | |
| | *unadjusted* | | *adjusted* | |
| above 1,500 altitude | -0.271*** | [-0.369,-0.173] | -0.252*** | [-0.339,-0.164] |
| | N=98,472 | | N=86,680 | |
| | **age range: 6–11 months** | | | |
| | *unadjusted* | | *adjusted* | |
| above 1,500 altitude | -0.276*** | [-0.395,-0.157] | -0.207*** | [-0.290,-0.123] |
| | N=105,197 | | N=93,354 | |
| | **age range: 12–23 months** | | | |
| | *unadjusted* | | *adjusted* | |
| above 1,500 altitude | -0.282*** | [-0.399,-0.164] | -0.170*** | [-0.243,-0.096] |
| | N=200,286 | | N=177,232 | |
| | **age range: 24–59 months** | | | |
| | *unadjusted* | | *adjusted* | |
| above 1,500 altitude | -0.291*** | [-0.394,-0.189] | -0.164*** | [-0.220,-0.107] |
| | N=560,344 | | N=493,415 | |

Data are regression coefficient with 95% CIs in brackets. Statistical significance denoted at * p < 0.05, ** p <0.01, *** p < 0.001. The estimates in 'unadjusted' column are based on unadjusted regression. The estimates in 'adjusted' column are based on adjusted regression that controls for child age (set of binary variables for each month) and sex, maternal age and education, household wealth and access to improved water and sanitation, binary variable capturing rural areas, and subnational region (highest administrative unit in each country) fixed effects.

© 2020 American Medical Association. All rights reserved. 

## eAppendix 4: Robustness to controlling for climate factors

Temperature and precipitation patterns differ in low and high altitude locations. For example, as altitude increases, average temperatures decrease. Our sub-national region fixed effects control for differences in climatic (and other) factors between sub-national regions. This leaves the possibility that climatic factors *within* sub-national regions are driving the association altitude and HAZ. To assess this possibility, we added additional controls to our main regression: average annual rainfall (in mm) in the cluster in 1981-2010 and average annual temperature (in C) in the cluster in 1981-2010. These climatic variables were calculated using University of East Anglia's (UEA) Climatic Research Unit (CRU) monthly climate data [12] and linked to the DHS data using GPS coordinates. These climate data could not be linked to all surveys or DHS clusters because of missing GPS coordinates. As a result, we lose 6.5 percent of the sample. As shown in eTable 8, the results remained remarkably robust indicating that the association between altitude and child HAZ was not driven by differences in climatic factors in low and high altitude locations – if anything the association gets stronger in absolute terms when we control for differences in climatic factors.

© 2020 American Medical Association. All rights reserved. 

**eTable 8: Regression results for the (unadjusted and adjusted) association between altitude and HAZ, after controlling for differences in climatic factors (temperature and rainfall)**

| | | | | |
|---|---|---|---|---|
| | **age range: 0–59 months** | | | |
| | *unadjusted* | | *adjusted* | |
| altitude (1,000 masl) | -0.130*** | [-0.182,-0.0775] | -0.207*** | [-0.248,-0.166] |
| | N = 901,586 | | N = 804,979 | |
| | **age range: 0–5 months** | | | |
| | *unadjusted* | | *adjusted* | |
| altitude (1,000 masl) | -0.113*** | [-0.160,-0.0657] | -0.196*** | [-0.271,-0.120] |
| | N = 92,157 | | N = 82,144 | |
| | **age range: 6–11 months** | | | |
| | *unadjusted* | | *adjusted* | |
| altitude (1,000 masl) | -0.142*** | [-0.197,-0.0867] | -0.244*** | [-0.313,-0.176] |
| | N = 98,725 | | N = 88,588 | |
| | **age range: 12–23 months** | | | |
| | *unadjusted* | | *adjusted* | |
| altitude (1,000 masl) | -0.142*** | [-0.200,-0.0846] | -0.231*** | [-0.276,-0.187] |
| | N = 187,702 | | N = 168,083 | |
| | **age range: 24–59 months** | | | |
| | *unadjusted* | | *adjusted* | |
| altitude (1,000 masl) | -0.126*** | [-0.182,-0.0698] | -0.193*** | [-0.243,-0.144] |
| | N = 523,002 | | N = 466,164 | |

Data are regression coefficient with 95% CIs in brackets. Statistical significance denoted at * $p < 0.05$, ** $p < 0.01$, *** $p < 0.001$. The estimates in ‘unadjusted’ column are based on unadjusted regression. The estimates in 'adjusted' column are based on adjusted regression that controls for child age (set of binary variables for each month) and sex, maternal age and education, household wealth and access to improved water and sanitation, binary variable capturing rural areas, and subnational region (highest administrative unit in each country) fixed effects. Additional controls include average annual rainfall (in mm) in the cluster in 1981-2010 and average annual temperature (in C) in the cluster in 1981-2010. The sample is restricted to DHS surveys for which climate data could be linked to.

© 2020 American Medical Association. All rights reserved. 

## eAppendix 5: Robustness to controlling for maternal stature

Low maternal height is found to be a risk factor for linear growth faltering and adverse birth outcomes [13,14]. It is possible that differences in maternal height could be a confounding factor that explains differences in child height between low and high altitude locations. However, maternal height captures both genetic and non-genetic factors [15]. Controlling for genetic differences between low and high altitude locations is desirable but if maternal height was influenced by the environment (i.e., altitude) in which the mother herself grew up, then it is not clear whether we should control for maternal height in the multivariable regression model. More generally, including variables that could be considered themselves as outcome variables are labelled as 'bad controls' in the statistics literature because their inclusion blurs the interpretation of the coefficient on the main independent variable (here: altitude) (see Angrist and Pischke 2009, p. 64-68).

Mindful of this conceptual ambiguity, we did not include maternal height as a control variable in the multivariable regression model reported in the main text. To explore sensitivity in this regard, we re-estimated the top panel of Table 1 ('All children') by adding maternal height to the regression model. eTable 9 shows that estimated coefficients were similar to those reported in Table 1 of the main text.

© 2020 American Medical Association. All rights reserved. 

**eTable 9: Regression results for the (unadjusted and adjusted) association between altitude and HAZ, after controlling for differences in maternal height**

| | | | | |
|---|---|---|---|---|
| | **age range: 0–59 months** | | | |
| | *unadjusted* | | *adjusted* | |
| altitude (1,000 masl) | -0.140*** | [-0.189,-0.0902] | -0.132*** | [-0.172,-0.0925] |
| | N=964,299 | | N=739,270 | |
| | **age range: 0–5 months** | | | |
| | *unadjusted* | | *adjusted* | |
| altitude (1,000 masl) | -0.111*** | [-0.155,-0.0674] | -0.160*** | [-0.213,-0.107] |
| | N=98,472 | | N=74,987 | |
| | **age range: 6–11 months** | | | |
| | *unadjusted* | | *adjusted* | |
| altitude (1,000 masl) | -0.147*** | [-0.200,-0.0951] | -0.172*** | [-0.217,-0.127] |
| | N=105,197 | | N=81,741 | |
| | **age range: 12–23 months** | | | |
| | *unadjusted* | | *adjusted* | |
| altitude (1,000 masl) | -0.149*** | [-0.204,-0.0944] | -0.160*** | [-0.200,-0.121] |
| | N=200,286 | | N=154,437 | |
| | **age range: 24–59 months** | | | |
| | *unadjusted* | | *adjusted* | |
| altitude (1,000 masl) | -0.140*** | [-0.193,-0.0863] | -0.113*** | [-0.162,-0.0632] |
| | N=560,344 | | N=428,105 | |

Data are regression coefficient with 95% CIs in brackets. Statistical significance denoted at * $p < 0.05$, ** $p < 0.01$, *** $p < 0.001$. The estimates in 'unadjusted' column are based on unadjusted regression. The estimates in 'adjusted' column are based on adjusted regression that controls for child age (set of binary variables for each month) and sex, maternal age and education, household wealth and access to improved water and sanitation, binary variable capturing rural areas, and subnational region (highest administrative unit in each country) fixed effects. Additional controls include maternal height (in cm).

© 2020 American Medical Association. All rights reserved. 

## eAppendix 6: Robustness to restricting the sample to children whose mother had lived in the same location at least since the conception

Children's heights in the DHS are measured at the time of the household interview. It could be that the location in which the child lived at the time of measurement was different to the location in which the child was born. Including these 'migrant' children in the sample could cause a bias to our 'altitude deficit' estimates if the altitude of the place of birth was different to the place of the DHS cluster in which the child resided at the time of the interview.

Fortunately, some DHS surveys collect information about the households' recent migration history. More specifically, the questionnaire asks "How long have you been living continuously in (*name of current city/town/village of residence*)?". We have this information for 699,714 children. Using the child's current age and the response to this question, we calculated that about 73 percent of the children (for whom we have this information) were measured in the same location they were conceived in (i.e., the number of years in current location ≥ child's age in years +1 year). In eTable 10, we restricted the sample to these children whose mother had lived in the same location at least since the conception. We see that coefficients are very similar to those reported in the top panel of Table 1 ('All children').

© 2020 American Medical Association. All rights reserved. 

**eTable 10: Regression results for the (unadjusted and adjusted) association between altitude and HAZ, after restricting the sample to children who have lived in the same location at least since the conception**

| | | | | |
|---|---|---|---|---|
| | **age range: 0–59 months** | | | |
| | *unadjusted* | | *adjusted* | |
| altitude (1,000 masl) | -0.123*** | [-0.177,-0.069] | -0.179*** | [-0.220,-0.137] |
| | N=509,908 | | N=452,587 | |
| | **age range: 0–5 months** | | | |
| | *unadjusted* | | *adjusted* | |
| altitude (1,000 masl) | -0.108*** | [-0.153,-0.063] | -0.171*** | [-0.228,-0.114] |
| | N=60,354 | | N=53,779 | |
| | **age range: 6–11 months** | | | |
| | *unadjusted* | | *adjusted* | |
| altitude (1,000 masl) | -0.124*** | [-0.180,-0.067] | -0.204*** | [-0.258,-0.150] |
| | N=67,790 | | N=60,935 | |
| | **age range: 12–23 months** | | | |
| | *unadjusted* | | *adjusted* | |
| altitude (1,000 masl) | -0.126*** | [-0.186,-0.065] | -0.180*** | [-0.224,-0.136] |
| | N=113,842 | | N=101,761 | |
| | **age range: 24–59 months** | | | |
| | *unadjusted* | | *adjusted* | |
| altitude (1,000 masl) | -0.125*** | [-0.185,-0.064] | -0.173*** | [-0.226,-0.120] |
| | N=267,922 | | N=236,112 | |

Data are regression coefficient with 95% CIs in brackets. Statistical significance denoted at * p < 0.05, ** p <0.01, *** p < 0.001. The estimates in 'unadjusted' column are based on unadjusted regression. The estimates in 'adjusted' column are based on adjusted regression that controls for child age (set of binary variables for each month) and sex, maternal age and education, household wealth and access to improved water and sanitation, binary variable capturing rural areas, and subnational region (highest administrative unit in each country) fixed effects. The sample is restricted to children who have lived in the same location at least since the conception.

© 2020 American Medical Association. All rights reserved. 

## eAppendix 7: Robustness to omitting low altitude countries from the sample

We had 18 countries in our sample that have no DHS clusters in high altitude locations (i.e., ≥ 1,500 masl). We conducted a sensitivity test in which replicated the top panel of Table 1 reported in the main text but dropped these 18 countries from the sample. eTable 11 shows the results based on this sub-sample. The coefficients are similar to those reported in Table 1.

© 2020 American Medical Association. All rights reserved. 

**eTable 11: Regression results for the (unadjusted and adjusted) association between altitude and HAZ, after restricting the sample to countries that have clusters above 1,500 masl**

| | | | | |
|---|---|---|---|---|
| | **age range: 0–59 months** | | | |
| | *unadjusted* | | *adjusted* | |
| altitude (1,000 masl) | -0.163*** | [-0.224,-0.102] | -0.165*** | [-0.208,-0.121] |
| | N=782,509 | | N=688,492 | |
| | **age range: 0–5 months** | | | |
| | *unadjusted* | | *adjusted* | |
| altitude (1,000 masl) | -0.107*** | [-0.156,-0.058] | -0.187*** | [-0.243,-0.131] |
| | N=77,284 | | N=67,547 | |
| | **age range: 6–11 months** | | | |
| | *unadjusted* | | *adjusted* | |
| altitude (1,000 masl) | -0.144*** | [-0.203,-0.086] | -0.214*** | [-0.264,-0.164] |
| | N=83,662 | | N=73,748 | |
| | **age range: 12–23 months** | | | |
| | *unadjusted* | | *adjusted* | |
| altitude (1,000 masl) | -0.166*** | [-0.232,-0.099] | -0.181*** | [-0.222,-0.140] |
| | N=160,267 | | N=141,104 | |
| | **age range: 24–59 months** | | | |
| | *unadjusted* | | *adjusted* | |
| altitude (1,000 masl) | -0.174*** | [-0.241,-0.107] | -0.147*** | [-0.198,-0.096] |
| | N=461,296 | | N=406,093 | |

Data are regression coefficient with 95% CIs in brackets. Statistical significance denoted at * p < 0.05, ** p <0.01, *** p < 0.001. The estimates in 'unadjusted' column are based on unadjusted regression. The estimates in 'adjusted' column are based on adjusted regression that controls for child age (set of binary variables for each month) and sex, maternal age and education, household wealth and access to improved water and sanitation, binary variable capturing rural areas, and subnational region (highest administrative unit in each country) fixed effects. The sample is restricted to countries that have clusters above 1,500 masl.

© 2020 American Medical Association. All rights reserved. 

## eAppendix 8: Robustness to excluding each country individually from the sample

It is possible that our results are driven by one individual country characterized by high mean altitude and low mean HAZ. To explore this possibility, we ran 59 adjusted regressions based a sample of children 0-59 months of age. In each regression, we omitted one country from the sample to assess the sensitivity of our coefficients. eTable 12 shows that the coefficients remain remarkably similar across the 59 sub-samples indicating that our results are not driven by one individual country. The largest negative coefficient is obtained when India is omitted from the sample (-0.206) and smallest negative coefficient is obtained when Guatemala is omitted from the sample (-0.147).

© 2020 American Medical Association. All rights reserved. 

**eTable 12: Regression results for the adjusted association between altitude and HAZ, excluding each country individually from the sample**

| Country omitted | coefficient | 95-% CI | N |
|---|---|---|---|
| Albania | -0.162*** | -0.205,-0.119 | 849,229 |
| Armenia | -0.162*** | -0.205,-0.119 | 849,112 |
| Angola | -0.163*** | -0.206,-0.120 | 844,398 |
| Azerbaijan | -0.163*** | -0.206,-0.120 | 846,984 |
| Bangladesh | -0.163*** | -0.206,-0.120 | 845,353 |
| Burkina Faso | -0.163*** | -0.206,-0.120 | 826,933 |
| Benin | -0.164*** | -0.207,-0.121 | 833,521 |
| Bolivia | -0.164*** | -0.209,-0.120 | 842,934 |
| Burundi | -0.162*** | -0.206,-0.119 | 841,288 |
| Congo Democratic Republic | -0.162*** | -0.205,-0.119 | 839,194 |
| Central African Republic | -0.163*** | -0.205,-0.120 | 848,297 |
| Cote d'Ivoire | -0.162*** | -0.205,-0.120 | 842,526 |
| Cameroon | -0.161*** | -0.204,-0.118 | 842,350 |
| Colombia | -0.158*** | -0.202,-0.113 | 834,967 |
| Dominican Republic | -0.162*** | -0.205,-0.119 | 838,132 |
| Egypt | -0.164*** | -0.207,-0.121 | 812,761 |
| Ethiopia | -0.165*** | -0.210,-0.120 | 828,608 |
| Gabon | -0.162*** | -0.205,-0.120 | 847,309 |
| Ghana | -0.163*** | -0.205,-0.120 | 837,766 |
| Guinea | -0.163*** | -0.205,-0.120 | 840,415 |
| Guatemala | -0.147*** | -0.189,-0.105 | 839,106 |
| Guyana | -0.162*** | -0.205,-0.120 | 849,127 |
| Honduras | -0.163*** | -0.205,-0.120 | 850,681 |
| Haiti | -0.157*** | -0.201,-0.114 | 833,133 |
| India | -0.206*** | -0.231,-0.180 | 618,896 |
| Jordan | -0.163*** | -0.205,-0.120 | 850,681 |
| Kenya | -0.162*** | -0.205,-0.118 | 840,720 |
| Cambodia | -0.163*** | -0.206,-0.120 | 835,466 |
| Comoros | -0.163*** | -0.206,-0.120 | 848,254 |
| Kyrgyz Republic | -0.164*** | -0.207,-0.121 | 846,683 |
| Liberia | -0.163*** | -0.206,-0.120 | 843,113 |
| Lesotho | -0.163*** | -0.206,-0.120 | 846,501 |
| Morocco | -0.163*** | -0.207,-0.120 | 845,114 |
| Moldova | -0.163*** | -0.205,-0.120 | 849,344 |
| Madagascar | -0.161*** | -0.205,-0.118 | 842,493 |
| Mali | -0.162*** | -0.205,-0.119 | 830,686 |

© 2020 American Medical Association. All rights reserved. 

| Country omitted | coefficient | 95-% CI | N |
|---|---|---|---|
| Myanmar | -0.162*** | -0.205,-0.119 | 846,508 |
| Malawi | -0.162*** | -0.205,-0.118 | 832,652 |
| Mozambique | -0.163*** | -0.206,-0.120 | 841,176 |
| Nigeria | -0.158*** | -0.202,-0.114 | 800,572 |
| Niger | -0.163*** | -0.206,-0.120 | 846,773 |
| Namibia | -0.163*** | -0.206,-0.120 | 842,185 |
| Nepal | -0.163*** | -0.208,-0.118 | 840,698 |
| Peru | -0.160*** | -0.216,-0.103 | 819,169 |
| Pakistan | -0.163*** | -0.206,-0.120 | 847,284 |
| Rwanda | -0.161*** | -0.204,-0.118 | 839,379 |
| Sierra Leone | -0.163*** | -0.206,-0.120 | 844,206 |
| Senegal | -0.163*** | -0.206,-0.120 | 833,595 |
| Swaziland | -0.163*** | -0.206,-0.120 | 848,661 |
| Chad | -0.162*** | -0.205,-0.119 | 831,512 |
| Togo | -0.163*** | -0.206,-0.120 | 846,978 |
| Tajikistan | -0.164*** | -0.208,-0.120 | 840,570 |
| Timor-Leste | -0.161*** | -0.205,-0.118 | 837,018 |
| Tanzania | -0.160*** | -0.204,-0.117 | 832,561 |
| Uganda | -0.161*** | -0.204,-0.118 | 836,715 |
| Yemen | -0.163*** | -0.205,-0.120 | 850,681 |
| South Africa | -0.162*** | -0.205,-0.119 | 849,605 |
| Zambia | -0.161*** | -0.204,-0.118 | 833,906 |
| Zimbabwe | -0.161*** | -0.204,-0.117 | 835,019 |

Note: Sample of children 0-59 months of age. Data are regression coefficient. Statistical significance denoted at * $p < 0.05$, ** $p < 0.01$, *** $p < 0.001$. The estimates are based on adjusted regression that controls for child age (set of binary variables) and sex, maternal age and education, household wealth and access to improved water and sanitation, binary variable capturing rural areas, and subnational region (highest administrative unit in each country) fixed effects.

© 2020 American Medical Association. All rights reserved. 

## eAppendix 9: Robustness to using stunting as the outcome variable

The results presented in the main text focused on mean HAZ scores. However, the altitude deficit also exists if we used stunting (HAZ<-2) as the outcome variable. eFigure 5 shows how stunting rates are higher among children residing in high altitudes (≥1,500 masl) populations compared to children residing in lower altitudes (<1,500 masl). According to eTable 13, a 1,000—masl increase in altitude is associated with a 4.6 %-point increase in stunting prevalence in children 0-59 months. Of note is that the estimated altitude deficit was negligible for children 0-5 months of age in the unadjusted regression (including eFigure 5) but the estimate increased substantially when we controlled for differences in socio-economic characteristics in the adjusted model.

© 2020 American Medical Association. All rights reserved. 

**eFigure 5: Stunting-age trajectories in <1,500 masl (gray line) and ≥1,500 masl (red line) (N = 964,299)**

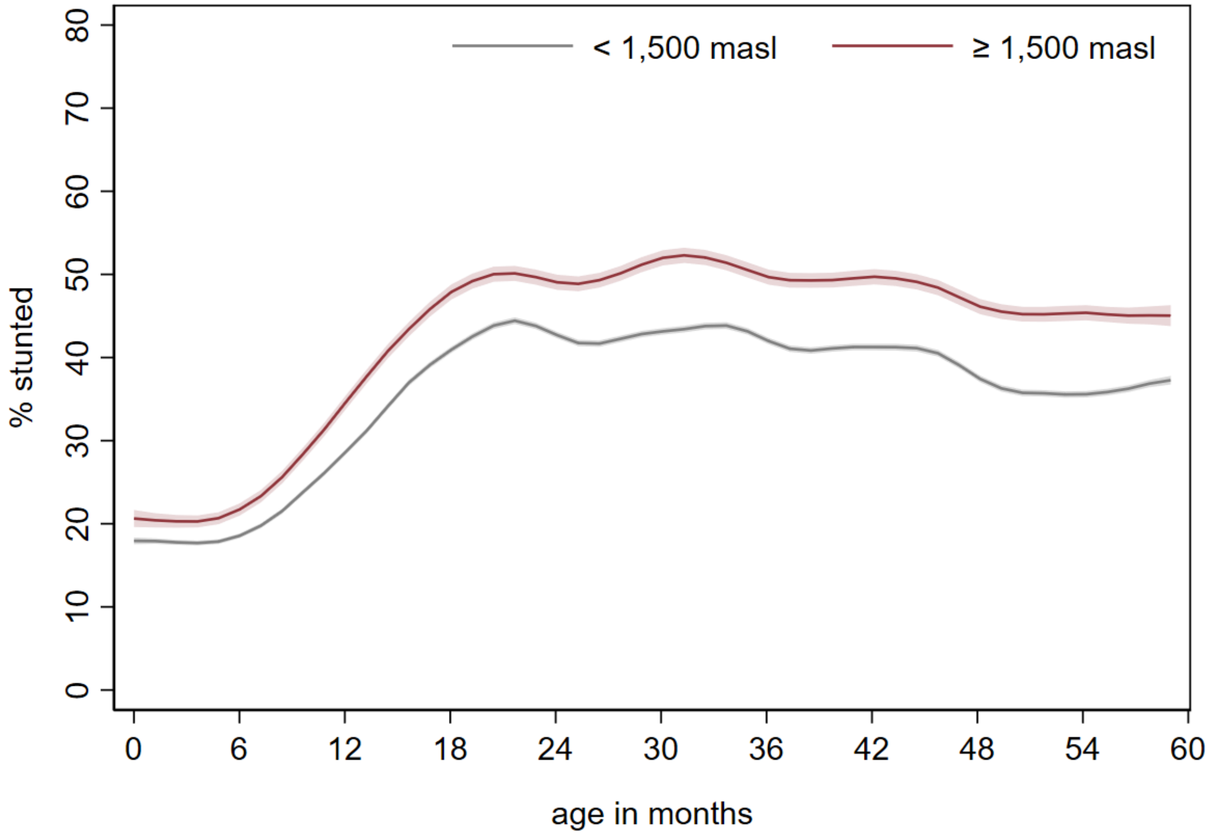


Note: See Figure 1 in the main text.

© 2020 American Medical Association. All rights reserved. 

**eTable 13: Regression results for the (unadjusted and adjusted) association between altitude and stunting (HAZ<-2)**

| | | | | |
|---|---|---|---|---|
| | **age range: 0-59 months** | | | |
| | *unadjusted* | | *adjusted* | |
| altitude (1,000 masl) | 0.0333*** | [0.019,0.048] | 0.0464*** | [0.034,0.059] |
| | N = 964,299 | | N = 850,681 | |
| | **age range: 0-5 months** | | | |
| | *unadjusted* | | *adjusted* | |
| altitude (1,000 masl) | 0.00793 | [-0.000,0.016] | 0.0341*** | [0.020,0.048] |
| | N = 98,472 | | N = 86,680 | |
| | **age range: 6-11 months** | | | |
| | *unadjusted* | | *adjusted* | |
| altitude (1,000 masl) | 0.0220*** | [0.011,0.033] | 0.0472*** | [0.032,0.062] |
| | N = 105,197 | | N = 93,354 | |
| | **age range: 12-23 months** | | | |
| | *unadjusted* | | *adjusted* | |
| altitude (1,000 masl) | 0.0357*** | [0.020,0.051] | 0.0509*** | [0.037,0.064] |
| | N = 200,286 | | N = 177,232 | |
| | **age range: 24-59 months** | | | |
| | *unadjusted* | | *adjusted* | |
| altitude (1,000 masl) | 0.0386*** | [0.022,0.055] | 0.0463*** | [0.032,0.061] |
| | N = 560,344 | | N = 493,415 | |

Note: Data are regression coefficient with 95% CIs in brackets. Statistical significance denoted at * p < 0.05, ** p <0.01, *** p < 0.001. The estimates in ‘unadjusted’ column are based on unadjusted regression. The estimates in 'adjusted' column are based on adjusted regression that controls for child age (set of binary variables for each month) and sex, maternal age and education, household wealth and access to improved water and sanitation, binary variable capturing rural areas, and subnational region (highest administrative unit in each country) fixed effects.

© 2020 American Medical Association. All rights reserved. 

**eReferences**


1. Black R, Allen L, Bhutta Z, et al. Maternal and Child Undernutrition Study Group: Maternal and child undernutrition 1-maternal and child undernutrition: global and regional exposures and health consequences. *The Lancet.* 2008;371:243-260.

2. UNICEF. *The State of the World's Children 1998.* New York: Oxford University Press; 1998.

3. WHO. *Indicators for assessing infant and young child feeding practices: part 1: definitions: conclusions of a consensus meeting held 6-8 November 2007 in Washington DC, USA.* Geneva: World Health Organization (WHO);2008.

4. Daelmans B, Dewey K, Arimond M. New and updated indicators for assessing infant and young child feeding. *Food and Nutrition Bulletin.* 2009;30(2_suppl2):S256-S262.

5. Working Group on Infant Young Child Feeding Indicators. Developing and validating simple indicators of dietary quality and energy intake of infants and young children in developing countries: summary of findings from analysis of 10 data sets. Washington D.C.: Food and Nutrition Technical Assistance Project (FANTA); 2006.

6. Arimond M, Ruel MT. Dietary diversity is associated with child nutritional status: evidence from 11 demographic and health surveys. *The Journal of Nutrition.* 2004;134(10):2579-2585.

7. Krasevec J, An X, Kumapley R, Bégin F, Frongillo EA. Diet quality and risk of stunting among infants and young children in low-and middle-income countries. *Maternal & child nutrition.* 2017;13:e12430.

8. WHO, UNICEF. Global Nutrition Monitoring Framework: operational guidance for tracking progress in meeting targets for 2025. Geneva: World Health Organization (WHO); 2018.

9. Center for International Earth Science Information Network - CIESIN - Columbia University. National Aggregates of Geospatial Data Collection: Population, Landscape, And Climate Estimates, Version 3 (PLACE III). Palisades, NY: NASA Socioeconomic Data and Applications Center (SEDAC); 2012.

10. Karra M, Subramanian S, Fink G. Height in healthy children in low-and middle-income countries: an assessment. *The American Journal of Clinical Nutrition.* 2016;105(1):121-126.

11. Royston P, Altman DG, Sauerbrei W. Dichotomizing continuous predictors in multiple regression: a bad idea. *Statistics in medicine.* 2006;25(1):127-141.

12. Harris I, Jones PD, Osborn TJ, Lister DH. Updated high-resolution grids of monthly climatic observations–the CRU TS3. 10 Dataset. *International journal of climatology.* 2014;34(3):623-642.


© 2020 American Medical Association. All rights reserved. 

13. Subramanian S, Ackerson LK, Smith GD, John NA. Association of maternal height with child mortality, anthropometric failure, and anemia in India. *JAMA.* 2009;301(16):1691-1701.

14. Addo OY, Stein AD, Fall CH, et al. Maternal height and child growth patterns. *The Journal of Pediatrics.* 2013;163(2):549-554. e541.

15. Silventoinen K. Determinants of variation in adult body height. *J Biosoc Sci.* 2003;35(02):263-285.

16. Angrist JD, Pischke J-S. *Mostly harmless econometrics: an empiricist's companion.* Princeton: Princeton University Press; 2009.


© 2020 American Medical Association. All rights reserved.
